# Ni^II^ and Cu^II^ complexes of a salen ligand bearing ferrocenes in its secondary coordination sphere†

**DOI:** 10.1039/d2ra07671c

**Published:** 2023-03-06

**Authors:** Rachel Sailer, Warren VandeVen, Kaeden Teindl, Linus Chiang

**Affiliations:** a Department of Chemistry, University of the Fraser Valley Abbotsford V2S 7M8 British Columbia Canada linus.chiang@ufv.ca; b Department of Chemistry, Simon Fraser University Burnaby V5A 1S6 British Columbia Canada

## Abstract

Herein, we report the synthesis, spectroscopic characterization and electrochemical investigation of the Ni^II^ and Cu^II^ complexes of a novel Sal ligand bearing two ferrocene moieties attached at its diimine linker, M(Sal)^Fc^. The electronic spectra of M(Sal)^Fc^ are near identical to its phenyl-substituted counterpart, M(Sal)^Ph^, indicating the ferrocene moieties exist in the secondary coordination sphere of M(Sal)^Fc^. The cyclic voltammograms of M(Sal)^Fc^ exhibit an additional two-electron wave in comparison to M(Sal)^Ph^, which is assigned to the sequential oxidation of the two ferrocene moieties. The chemical oxidation of M(Sal)^Fc^, monitored by low temperature UV-vis spectroscopy, supports the formation of a mixed valent Fe^II^Fe^III^ species followed by a bis(ferrocenium) species upon sequential addition of one and two equivalents of chemical oxidant. The addition of a third equivalent of oxidant to Ni(Sal)^Fc^ yielded intense near-IR transitions that are indicative of the formation of a fully delocalized Sal-ligand radical (Sal˙), while the same addition to Cu(Sal)^Fc^ yielded a species that is currently under further spectroscopic investigation. These results suggest the oxidation of the ferrocene moieties of M(Sal)^Fc^ does not affect the electronic structure of the M(Sal) core, and these are thus in the secondary coordination sphere of the overall complex.

## Abstract 2

The synthesis and characterization of two novel Ni^II^ and Cu^II^ salen complexes containing ferrocene moieties in their secondary coordination sphere are described herein.

## Graphical abstract

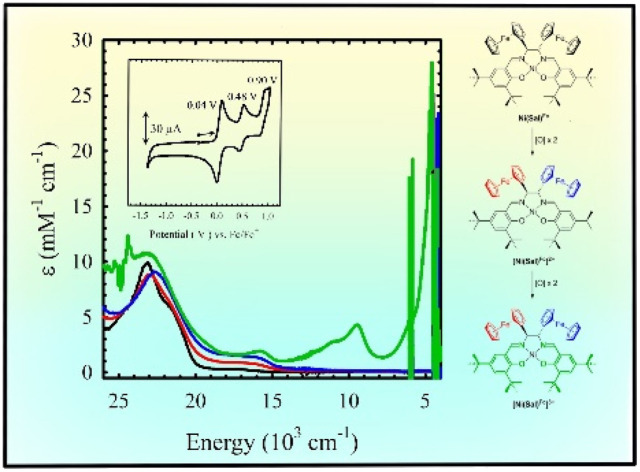

## Introduction

Salen, a common abbreviation for the tetradentate N_2_O_2_ bis(iminophenoxide) ligand scaffold (referred to as Sal herein), is a highly popular ligand scaffold due to the ease of its synthesis and the ability to robustly coordinate transition metal ions in a variety of oxidation states.^1–6^ The Sal ligand scaffold is a redox active ligand: M(Sal) complexes can undergo metal- or ligand- oxidation depending on the relative energies of the metal and ligand frontier orbitals. The locus of oxidation is highly sensitive to experimental conditions such as solvent, presence of extraneous ligands and even temperature, illustrating the similarity in energy between the two oxidized states.^7–13^ For example, the oxidation of Ni^II^(Sal) yields a ligand-oxidized species [Ni^II^(Sal)˙]^+^ in non-coordinating solvents such as CH_2_Cl_2_,^14,15^ which is converted to a metal-oxidized species [Ni^III^(Sal)(L)_2_]^+^ in the presence of coordinating solvents or extraneous ligands, where the shift in locus of oxidation in accompanied by concomitant axial ligation.^14–18^ The locus of oxidation is also dependant on the substituents on the Sal scaffold: oxidation of M(Sal)^R^, where R is an electron donating substituent at the distal *para*-position of the phenoxide moiety, generally yields ligand oxidized products.^14,15,19–25^ On the other hand, metal oxidized products are observed if R is an electron withdrawing substituent at the same position.^21,22,26^ Similarly, the oxidation of Cu^II^(Sal) yields metal- ([Cu^III^(Sal)]^+^) and ligand-oxidized ([Cu^II^(Sal˙)]^+^) products that can exist in a temperature dependant spin equilibrium.^19,20^

The N_2_O_2_ donor atoms of the Sal ligand scaffold, which are directly ligated to metal ions upon coordination, constitute the primary coordination sphere of M(Sal). Beyond this coordination sphere is the secondary coordination sphere, consisting of groups that are not directly coordinated to the metal center. An archetypal example is the enzyme Ni–Fe carbon monoxide dehydrogenase, which contains non-coordinated, Brønsted basic histidine and lysine amino acid residues in its secondary coordination sphere that facilitates the reduction of a bound CO_2_ substrate *via* hydrogen bonding.^27^ Inspired by this, the incorporation of similar Brønsted acidic and basic groups in the secondary coordination spheres of inorganic complexes have received significant research interest in recent years.^28–31^

**Chart 1:**
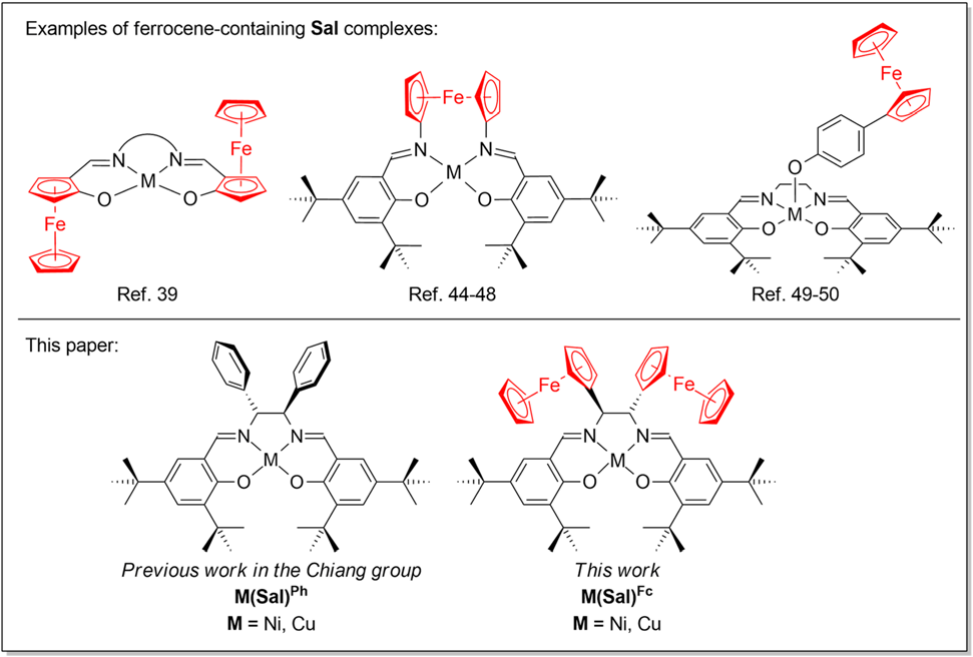
Ferrocene-containing Sal complexes.

In general, the Sal ligand platform is capable of supplying two electron equivalents upon its oxidation, although further oxidizing equivalents are possible when electron-donating substituents are incorporated on the ligand scaffold. Our research group is specifically interested in incorporating redox active moieties, such as ferrocene, onto the already redox active Sal ligand scaffold. Ferrocene moieties have been introduced to redox-active ligand scaffolds, resulting in complexes that exhibit rich electrochemical behaviour.^32–35^ Ferrocene moieties have been incorporated into M(Sal) complexes, most commonly *via* the aromatic system of Sal,^36–43^ diamine linker,^44–48^ or axial coordination of a ferrocene-containing ligand (Chart 1).^49,50^ However their incorporation into the secondary coordination sphere of Sal*via* the sp^3^-carbon of the diamine linker have been far less explored.^51^ Furthermore, the electronic spectroscopy and electrochemical behaviors of these compounds have yet to be reported. To this end, this paper will describe the synthesis, spectroscopic characterization and electrochemical studies of a Ni^II^ and Cu^II^ complexes of a novel Sal ligand scaffold in which two ferrocene moieties are incorporated into its secondary coordination sphere *via* its diimine linker (Chart 1). The formation of their corresponding oxidized forms using a suitable chemical oxidant were investigated using low temperatures UV-vis spectroscopy.

## Experimental

### Materials and methods

All chemicals used were of the highest grade available and were used as received. Schiff base 1 was synthesized based on a previous report.^51^ The tris(2,4-dibromophenyl)aminium hexafluoroantimonate oxidant (N(C_6_H_3_Br_2_)_3_SbF_6_, *E*_1/2_ = 1.14 V *vs.* Fc/Fc^+^ in MeCN)^52,53^ was prepared from commercially available triphenylamine by reported procedures.^17,54^^1^H and ^13^C NMR spectra were collected at Trinity Western University on a Bruker ASCEND III 400 MHz with Bruker AVANCE III 400 MHz instrument running the TopSpin 3.1.6 program. Mass spectrometry data (electrospray ionization (ESI) positive ion or ESI negative ion) were collected at Simon Fraser University on an Agilent 6210 TOF ESI-MS instrument. Elemental analyses (C, H, N) were performed at the University of British Columbia on a Thermo Flash 2000 elemental analyzer. Electronic spectra were collected on a Cary 50 spectrophotometer using a custom designed immersion fiber-optic probe with a path length of 1 mm (Hellma Inc.). Temperatures were maintained during data collection using a dry ice/acetone bath (−78 °C). Cyclic voltammetry (CV) data were collected on a CHI-630E potentiostat connected to a three electrode voltammetry cell (Ag/AgCl wire reference electrode, glassy carbon working electrode, and a Pt auxiliary electrode) with tetra-*n*-butylammnoiumperchlorate (^*n*^Bu_4_NClO_4_, 0.1 M) as supporting electrolyte. Cobaltaceniumhexafluorophosphate (*E*_1/2_ = −1.34 V *vs.* Fc/Fc^+^ in 0.1 M ^*n*^Bu_4_NClO_4_ in CH_2_Cl_2_) were used as internal standards.

### Oxidation protocol

Under an Ar atmosphere at 195 K, 1.0 mL of a CH_2_Cl_2_ solution of the metal complex (10.0 mM) was added to 10.0 mL of CH_2_Cl_2_. Monitored by UV-vis spectroscopy, a saturated solution of N(C_6_H_3_Br_2_)_3_SbF_6_ was added in 100 μL aliquots to yield the corresponding one-electron oxidized species.

### Synthesis

#### H_2_(Sal)^Fc^

To a clear red solution of Schiff base 1 (1.9926 g, 3.131 mmol) in THF (30 mL) was added 37% aqueous HCl (6 mL, 37%), and the solution stirred at room temperature for 3 hours. The resulting dark yellow precipitate was collected by vacuum filtration, washed with THF, and dried under vacuum to afford a pale-yellow powder that is presumably the diammonium salt (1.347 g, yield: 88%). This material was used in the subsequent steps immediately without purification. A portion of this salt (0.1000 g, 0.200 mmol) was suspended in methanol (5 mL), where triethylamine (0.18 mL, 1.29 mmol) and 3,5-di-*tert*-butylsalicylaldehyde (0.0935 g, 0.399 mmol) were added sequentially. The resulting reaction mixture became orange and clear immediately, which was left stirring at room temperature overnight. The resulting bright yellow precipitate was collected *via* vacuum filtration and washed with cold EtOH to yield H_2_(Sal)^Fc^ as a fluffy light yellow solid (0.1162 g, yield: 68%). ^1^H NMR (CDCl_3_, 400 MHz): *δ* = 13.82 (s, 2H, OH), 8.27 (s, 2H, imine-H), 7.34 (d, 2H, Ar–H), 6.95 (d, 2H, Ar–H), 4.28 (m, 2H, Fc-H), 4.13 (m, 2H, Fc-H), 4.09 (s, 2H, sp^3^-CH), 3.99 (m, 2H, Fc-H), 3.95 (s, 10H, Fc-H), 3.72 (m, 2H, Fc-H), 1.48 (s, 18H, *t*Bu-H), 1.23 (s, 18H, tBu-H). ^13^C NMR (CDCl_3_, 100 MHz): *δ* = 166.4 (N=*C*H), 158.2 (Ar–*C*), 140.1 (Ar–*C*), 136.6 (Ar–*C*), 127.2 (Ar–*C*), 126.2 (Ar–*C*), 117.7 (Ar–*C*), 88.8 (Fc-*C*C), 75.9 (N–*C*H-Fc), 69.4 (Fc-*C*H), 68.7 (Fc-*C*H), 68.1 (unsubstituted Fc-*C*H), 67.0 (Fc-*C*H), 66.9 (Fc-*C*H), 35.2 (*C*-(CH_3_)_3_), 34.2 (*C*-(CH_3_)_3_), 31.6 (C-(*C*H_3_)_3_), 29.6 (C-(*C*H_3_)_3_). MS (ESI): Found (*m*/*z* (%)): 861.3733 (100); calculated (*m*/*z* (%)): 861.3739 (100) [H_2_(Sal)^Fc^ + H^+^]^+^. Elemental analysis calculated (%) for H_2_(Sal)^Fc^: C: 72.56, H: 7.49, N: 3.25; Found (%) C: 72.60, H: 7.37, N: 3.23.

#### Ni(Sal)^Fc^

To a solution of H_2_(Sal)^Fc^ (0.1000 g, 0.12 mmol) Et_2_O (1 mL) was added a solution of Ni(OAc)_2_·4H_2_O (0.0289 g, 0.12 mmol) in MeOH (1 mL), which was left stirring overnight. A dark amber coloured solution with brown precipitate remained, where the solvent was evaporated *via* rotary evaporation to yield a yellow-brown powdered product. This crude material was purified by silica gel column chromatography using a 3 : 1 v/v pentane and dichloromethane as eluent (*R*_f_ = 0.70) to yield a dark brown powder of Ni(Sal)^Fc^ (0.017 g, 16%). ^1^H NMR (400 MHz, CDCl_3_): *δ* 7.54 (s, 1H, imine H), 7.31 (d, 1H, aromatic H), 6.91 (d, 1H, aromatic H), 4.80 (s, 1H, CH), 4.39 (s, 1H, Fc H), 4.27 (s, 6H, Fc H), 1.40 (s, 9H, *t*Bu H), 1.30 (s, 9H, *t*Bu H). ^13^C NMR (CDCl_3_, 100 MHz): *δ* = 163.6 (N=*C*H), 162.4 (Ar–*C*), 140.5 (Ar–*C*), 135.9 (Ar–*C*), 129.2 (Ar–*C*), 125.7 (Ar–*C*), 119.7 (Ar–*C*), 91.9 (Fc-*C*C), 75.4 (N–*C*H-Fc), 69.1 (Fc-*C*H), 68.4 (Fc-*C*H), 68.1 (Fc-*C*H), 66.5 (Fc-*C*H), 35.9 (*C*-(CH_3_)_3_), 33.9 (*C*-(CH_3_)_3_), 31.4 (C-(*C*H_3_)_3_), 29.7 (C-(*C*H_3_)_3_). MS (ESI): Found (*m*/*z* (%)): 917.2946 (100); calculated (*m*/*z* (%)): 917.2925(100) (917.2946 (66) [Ni(Sal)^Fc^ + H^+^]^+^, 916.2858 (34) [Ni(Sal)^Fc^ − e^−^]^+^). Elemental analysis calculated (%) for Ni(Sal)^Fc^: C: 68.08, H: 6.81, N: 3.05; Found (%) C: 68.51, H: 6.93, N: 2.84.

#### Cu(Sal)^Fc^

A solution of H_2_(Sal)^Fc^ (0.1000 g, 0.12 mmol) in Et_2_O (2 mL) was added a solution of Cu(OAc)_2_·H_2_O (0.0232 g, 0.12 mmol) in MeOH (2 mL), which was left stirring overnight. The murky green solution that remained was concentrated *in vacuo* to yield a green powder. This crude material was purified by silica gel column chromatography using 3 : 1 v/v pentane and dichloromethane as eluent (*R*_f_ = 0.71) to yield a dark green powder of Cu(Sal)^Fc^ (0.079 g, 74%). MS (ESI): Found (*m*/*z* (%)): 922.2880 (100); calculated (*m*/*z* (%)): 922.2866 (100) (922.2879 (87) [Cu(Sal)^Fc^ + H^+^]^+^, 921.2801 (13) [Cu(Sal)^Fc^ − e^−^]^+^). Elemental analysis calculated (%) for Cu(Sal)^Fc^: C: 67.72, H: 6.78, N: 3.04; Found (%) C: 67.89, H: 6.68, N: 2.88.

## Results and discussion

### Synthesis of ligands and complexes

**Scheme 1:**
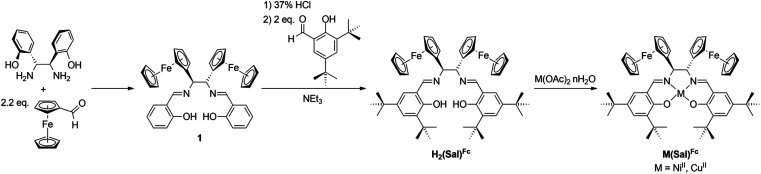
Synthetic scheme of ligand H_2_(Sal)^Fc^ and their corresponding complexes M(Sal)^Fc^.

Ligand H_2_(Sal)^Fc^ was synthesized in three steps from commercially available starting materials. First, a diaza-Cope rearrangement reaction between (1*R*,2*R*)-1,2-bis(2-hydroxyphenyl)-1,2-diaminoethane and 2.2 equivalents of ferrocenecarboxaldehyde yields Schiff-base 1.^51^ While 1 is a ferrocene-containing Sal ligand, the lack of *ortho*- and *para*-substituents relative to the phenol oxygen is envisaged to produce less stable oxidized products, as unsubstituted M(Sal) complexes are known to undergo decomposition *via* oxidative radical polymerization at these positions.^55–59^ As such, 1 was hydrolyzed under acidic conditions, then condensed with 2 equivalents of 3,5-di-*tert*-butylsalicylaldehyde in the presence of an excess of NEt_3_ to afford the target ligand H_2_(Sal)^Fc^. Equimolar portions of H_2_(Sal)^Fc^ and the appropriate metal(II) acetate reagent were reacted to yield Ni(Sal)^Fc^ and Cu(Sal)^Fc^ (Scheme 1). All attempts to obtain single crystals of M(Sal)^Fc^ for X-ray diffraction analysis using column purified material resulted in oils due to their high solubility in nearly all organic solvents.

### Characterization of M(Sal)^R^

*In lieu* of a solid state structure for M(Sal)^Fc^, a combination of solution-based high resolution ESI mass spectrometry, NMR spectroscopy and theoretical DFT calculations were employed to establish their structures. H_2_(Sal)^Fc^ exhibit ESI-MS signals at *m*/*z* = 861.3733, as expected for its corresponding mono-protonated [H_2_(Sal)^Fc^ + H^+^]^+^ species (Fig. S1†). Similar mono-protonated species were observed in the mass spectra of M(Sal)^Fc^ (*m*/*z* (%): [Ni(Sal)^Fc^ + H^+^]^+^: 917.2946 (66); [Cu(Sal)^Fc^ + H^+^]^+^: 922.2879 (87)). However, the observed isotope pattern can only be accurately modeled when a minor species at *ca.* 1 *m*/*z* unit less was considered both M(Sal)^Fc^, which can be attributed to a mono-oxidized species (*m*/*z* (%): [Ni(Sal)^Fc^ − e^−^]:^+^: 916.2858 (34); [Cu(Sal)^Fc^ − e^−^]^+^: 921.2801 (13); Fig. S2 and S3†). Taken together, these data suggest that both M(Sal)^Fc^ are 1 : 1 metal to Sal complexes that forms with the concomitant deprotonation of H_2_(Sal)^Fc^.

**Fig. 1:**
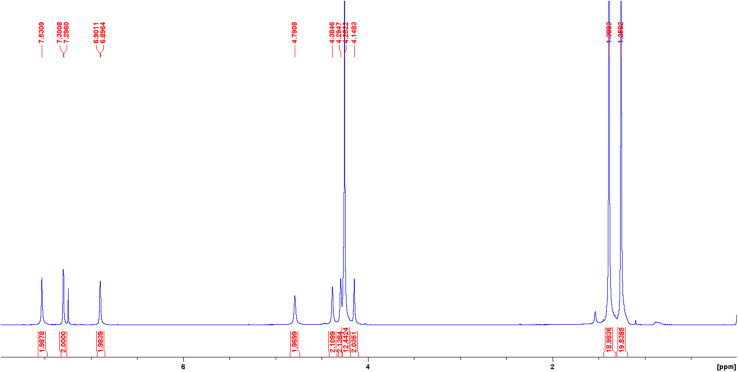
^1^H NMR spectrum of Ni(Sal)^Fc^ recorded in CDCl_3_ at 298 K.

The ^1^H NMR spectra of both diamagnetic H_2_(Sal)^Fc^ and Ni(Sal)^Fc^ reveal their *C*_2_-symmetry on the NMR timescale (Fig. 1 and S4–S6†). The imine (*δ* = 7.5–8.5 ppm), phenol/phenolate (*δ* = 6.9–7.4 ppm) and *tert*-butyl (*δ* = 1.2–1.5 ppm) signals are consistent with those of H_2_(Sal)^Ph^ and Ni(Sal)^Ph^.^60^ Four signals were observed in the *δ* = 4–5 ppm region, which can be assigned to the proton environments of the monosubstituted η^5^-C_5_H_4_ ring due to their diastereotopic nature as they are adjacent to the stereogenic center on the diimine linker.^61^^1^H–^1^H COSY NMR experiments confirm this assignment by revealing the coupling between the protons on the monosubstituted η^5^-C_5_H_4_ ring, while ^1^H–^13^C HSQC NMR experiments demonstrate the coupling between these protons and the aromatic carbon signals (Fig. S7–S13†). The remaining signals that do not exhibit coupling to the protons of the monosubstituted η^5^-C_5_H_4_ ring are then assigned to the protons on the unsubstituted η^5^-C_5_H_5_ aromatic system and the proton on the diimine linker.

The structure of M(Sal)^Fc^ was examined *via* DFT calculations using the B3LYP/6–31g* functional/basis set combination and a PCM solvent model, parameters that have been used to investigate similar M(Sal) compounds. The optimized structures of M(Sal)^Fc^ both exhibit two ferrocene moieties on the diimine linker separated by large dihedral angles that results from their occupation of trans, diaxial sites (Ni(Sal)^Fc^: 158.2°; Cu(Sal)^Fc^: 159.8°, Fig. S14 and S15†). This configuration has been observed previously *via* crystallography for structurally similar compounds in which two ferrocene moieties are also attached on a diimine linker, and is expected due to their steric bulk.^51^ Both M(Sal)^Fc^ exhibit calculated coordinative bonds that are within 0.03 Å to their M(Sal)^Ph^ counterpart under the same computational parameters (Table S1†). In addition, the singly occupied molecular orbitals (SOMO) of Cu(Sal)^Fc^ consist predominantly of a d_*x*^2^–*y*^2^_ orbital with spin covalency with the coordinating atoms, which is expected for a square planar, d^9^, Cu^II^ complex (Fig. S16†). The predicted spin density on the Cu^II^ center of Cu(Sal)^Fc^ is identical to Cu(Sal)^Ph^ at *ca.* 57%.^21^ Overall, these results suggest that the two ferrocene moieties in the secondary coordination sphere of M(Sal)^Fc^ does not significantly affect the tetradentate and square planar geometry around the Ni^II^ and Cu^II^ centers afforded by the primary coordination sphere in M(Sal)^Fc^.

### Electronic spectroscopy of neutral M(Sal)^R^

**Fig. 2:**
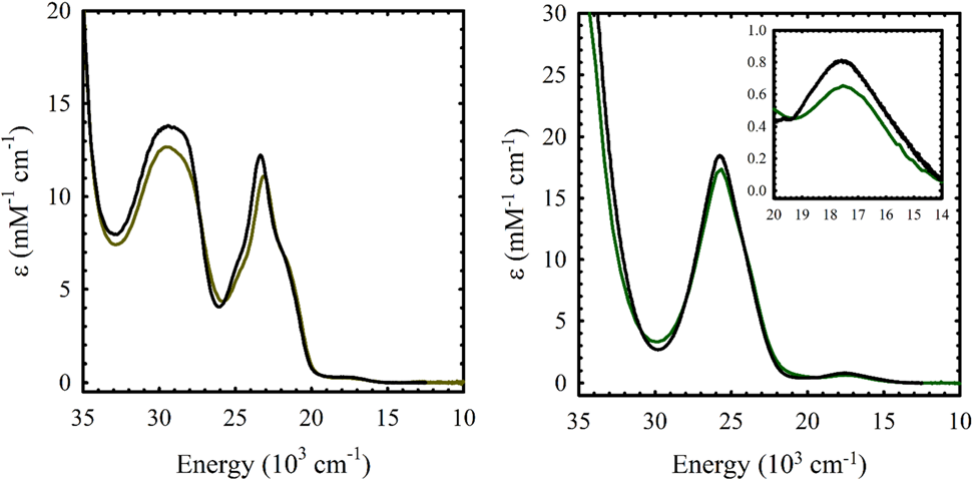
UV-vis spectra of *ca.* 0.1 mM solutions of (a) Ni(Sal)^Fc^ (left, brown) *vs.*Ni(Sal)^Ph^ (left, black) and (b) Cu(Sal)^Fc^ (right, green) *vs.*Cu(Sal)^Ph^ (right, black) in CH_2_Cl_2_. (Inset) Magnified view of the d–d transitions.

The electronic absorption spectrum of M(Sal)^Fc^ is nearly identical to its M(Sal)^Ph^ counterpart in CH_2_Cl_2_ (Fig. 2 and Table 1). A series of charge transfer transitions above 20 000 cm^−1^ and weak d–d transitions below 18 000 cm^−1^, typical of square-planar, d^8^ Ni^II^ bis-phenoxide salen complexes, were observed for Ni(Sal)^Fc^.^15,19,26^ On the other hand, an intense charge transfer transition above 25 000 cm^−1^ and a d–d transition above 17 300 cm^−1^ were observed for Cu(Sal)^Fc^, typical of square-planar, d^9^ Cu^II^ bis-phenoxide salen complexes.^19,20,26^ The d–d transitions of Cu(Sal)^Fc^, an indicator of ligand field strength, is observed at similar energies to its Cu(Sal)^Ph^ counterpart (*E*(Cu(Sal)^Fc^): 17 500 cm^−1^; *E*(Cu(Sal)^Ph^): 17 600 cm^−1^). This suggests the two ferrocene moieties on the diimine linker only minimally affects the primary coordination sphere afforded by the N_2_O_2_ donor atoms of the Sal scaffold, and thus exists in the secondary coordination sphere of the complex.

**Table 1:** UV-vis spectroscopy data of M(Sal)^R^ in CH_2_Cl_2_

Compound	UV-vis [*E*, 10^3^ cm^−1^ (*ε*, mM^−1^ cm^−1^)]
Ni(Sal)^Ph^	29.5 (13.8), 23.4 (12.2), 17.5 (0.3)
Ni(Sal)^Fc^	29.5 (12.7), 23.1 (11.1), 17.5 (0.3)
Cu(Sal)^Ph^	25.8 (18.3), 17.6 (0.8)
Cu(Sal)^Fc^	25.7 (17.3), 17.5 (0.6)

### Electrochemistry

**Fig. 3:**
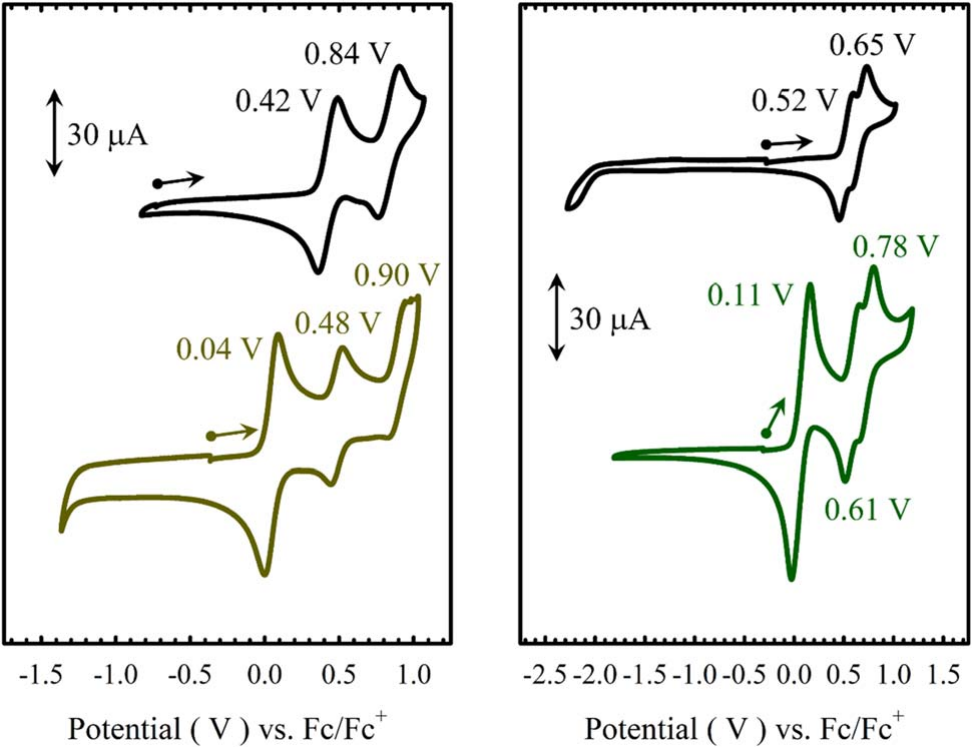
Cyclic voltammograms of *ca.* 1.5 mM solutions in CH_2_Cl_2_ of (a) Ni(Sal)^Ph^ (left, black) *vs.*Ni(Sal)^Fc^ (left, brown), and (b) Cu(Sal)^Ph^ (right, black) *vs.*Cu(Sal)^Fc^ (right, green).

The electrochemical behaviours of M(Sal)^Fc^ in CH_2_Cl_2_ were probed by cyclic voltammetry (CV) in the presence of tetra-*n*-butylammonium perchlorate (^*n*^Bu_4_NClO_4_, 0.1 M) as supporting electrolyte (Fig. 3 and Table 2). Three quasi-reversible oxidative waves are observed for both M(Sal)^Fc^ (Ni(Sal)^Fc^: 0.04 V, 0.48 and 0.90 V *vs.* Fc/Fc^+^; Cu(Sal)^Fc^: 0.11 V, 0.61 and 0.78 V *vs.* Fc/Fc^+^). The least positive redox couple is assigned to the oxidation of the two ferrocene moieties on the basis of its similarity in *E*_1/2_ to the Fc/Fc^+^ reference couple and its absence in the CV of the corresponding M(Sal)^Ph^ complexes.^60^ The current response of this redox couple is nearly twice that of the remaining couples on CV. This suggests the successive oxidation of the two ferrocene moieties first affords a one-electron oxidized, charge localized, mixed valence Fe^II^Fe^III^ species followed by a two-electron oxidized Fe^III^Fe^III^ species under these experimental conditions, in which the two ferrocenes are electronically isolated. This is in contrast to the electrochemical behaviour of biferrocenes that are connected such that the iron centers are closely associated or *via* a conjugated linker, both of which allow the two iron centers to interact electronically.^62–64^ The formation of a mixed valence species is expected for one-electron oxidized [M(Sal)^Fc^]^+^, as the two ferrocene moieties are connected *via* two unsaturated sp^3^-hybridized carbons,^62,65^ and are likely well separated due to the steric bulk afforded by the ferrocene moieties.^51^

**Table 2:** Redox potentials (*V*) of M(Sal)^R^ complexes *vs.* Fc^+^/Fc in CH_2_Cl_2_^a^^,^^b^

Compound	*E*_1/2_^1^	*E*_1/2_^2^	*E*_1/2_^3^
Ni(Sal)^Ph^	0.42 (0.13)	0.84 (0.14)	—
Ni(Sal)^Fc^	0.04 (0.09)	0.48 (0.08)	0.90 (0.12)
Cu(Sal)^Ph^	0.52 (0.15)	0.65 (0.16)	—
Cu(Sal)^Fc^	0.11 (0.12)	0.61 (0.14)	0.78 (0.15)

a Peak-to-peak difference (*E*^a^_p_ − *E*^c^_p_) are provided in brackets (V).

b Peak-to-peak difference for the Cp_2_Co^+^/Cp_2_Co at 298 K is 0.08 V.

The remaining two couples of M(Sal)^Fc^, *E*_1/2_^2^ and *E*_1/2_^3^, are thus assigned to the same oxidative events as those observed for M(Sal)^Ph^.^21,60^ The quasi-reversible nature of these couples suggests that oxidation of the ferrocene moieties does not affect the chemical stability of the electrogenerated species on the timescale of the electrochemistry experiment. The *E*_1/2_^2^ and *E*_1/2_^3^ of M(Sal)^Fc^ are slightly anodically shifted in comparison to the *E*_1/2_^1^ and *E*_1/2_^2^ of M(Sal)^Ph^, which can be attributed to the dicationic nature of the overall complex before the next oxidation occurs (*i.e.*[M(Sal)^Fc^]^2+^ → [M(Sal)^Fc^]^3+^*vs.*M(Sal)^Ph^ → [M(Sal)^Ph^]^+^). In addition, the difference in potentials between *E*_1/2_^2^ and *E*_1/2_^3^ of Ni(Sal)^Fc^ is identical to *E*_1/2_^1^ and *E*_1/2_^2^ of Ni(Sal)^Ph^ (Δ*E*_1/2_ = 0.42 V). The magnitude of Δ*E*_1/2_ between the first two oxidative couples of Ni(Sal) compounds have been correlated to the degree of delocalization of ligand radical formed upon one-electron oxidation.^15^ This suggests that [Ni(Sal)^Fc^]^3+^ and [Ni(Sal)^Ph^]^+^ must exhibit similar Sal˙ electronic structures. Taken together, these data support the ferrocene moieties are indeed in the secondary coordination sphere of M(Sal)^Fc^ as their oxidation does not affect the subsequent oxidation of M(Sal) significantly (Table 2).

### Chemical oxidation of M(Sal)^Fc^

**Fig. 4:**
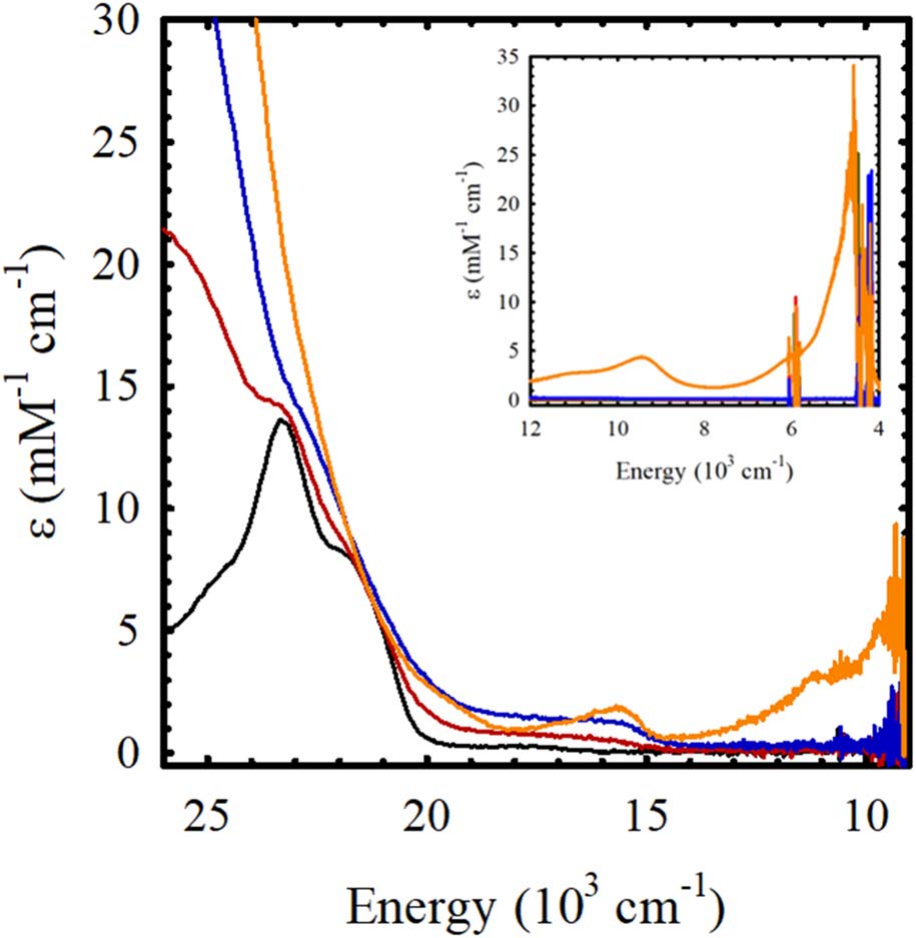
Sequential chemical oxidation of Ni(Sal)^Fc^ (black) to its one-electron oxidized (red), two-electron oxidized (blue), and three-electron oxidized (orange) species. (Inset) Near-IR region. Conditions: 0.4 mM Ni(Sal)^Fc^ in CH_2_Cl_2_, 195 K.

Chemical oxidation of Ni(Sal)^Fc^ by incremental addition of two equivalents of the aminyl oxidant N(C_6_H_3_Br_2_)_3_SbF_6_ (*E* = 1.14 V *vs.* Fc/Fc^+^ in CH_2_Cl_2_) in CH_2_Cl_2_ at 195 K leads to the formation of a broad envelope of weak transitions between 15 000–20 000 cm^−1^ (*ε* = 1 – 2 mM^−1^ cm^−1^), that can be attributed to the formation of a ferrocenium-containing species (Fig. 4 and Table 3). The doubling in intensity of these transition, alongside the lack of near-IR transitions upon the addition of a second equivalent of oxidant strongly supports the formation of a charge-localized mono(ferrocenium) (Fe^II^Fe^III^) species, followed by a bis(ferrocenium) (Fe^III^Fe^III^) species upon the addition of the first two equivalents of oxidant. The addition of a third equivalent of the aminyl oxidant to Ni(Sal)^Fc^ yields NIR transitions at 9400 cm^−1^ (*ε* = 4.3 mM^−1^ cm^−1^) and 4600 cm^−1^ (*ε* = 23.5 mM^−1^ cm^−1^) that are demonstrative of the oxidation of Sal to a fully delocalized [Sal˙]^+^ ligand radical.^15^ These results are fully consistent with the electrochemical data of Ni(Sal)^Fc^, where the ferrocene-based oxidative couple at 0.04 V *vs.* Fc/Fc^+^ exhibits minimal separation, suggesting the sequential formation of a mixed-valent ferrocene-ferrocenium species followed by a bis(ferrocenium) species.^66,67^ The anodic wave observed at 0.48 V *vs.* Fc/Fc^+^, attributed to Sal oxidation, is separated from the next anodic wave by 0.42 V, indicates the formation of a fully delocalized Sal˙ ligand radical (*vide supra*).

**Table 3:** UV-vis spectroscopy data of the chemical oxidation M(Sal)^Fc^ in CH_2_Cl_2_

Compound	UV-vis [*E*, 10^3^ cm^−1^ (*ε*, mM^−1^, cm^−1^)]
Ni(Sal)^Fc^	23.1 (9.9), 17.5 (0.3)
[Ni(Sal)^Fc^]^+^	23.0 (8.8), *ca.* 17.4 (br, 0.8)
[Ni(Sal)^Fc^]^2+^	22.6 (9.1), *ca* 17.4 (br, 1.6)
[Ni(Sal)^Fc^]^3+^	23.1 (10.6), 15.7 (1.8), 9.4 (4.3), 4.6 (23.5)

Similarly, addition of two equivalents of aminyl oxidant to Cu(Sal)^Fc^ yields weak transitions between 15 000–20 000 cm^−1^ (*ε* = 1 – 2 mM^−1^ cm^−1^) that can be attributed to the formation of a bis(ferrocenium) species (Fig. S17†). However, the addition of a third equivalent of the aminyl oxidant to Cu(Sal)^Fc^ yields broad and weak transitions at 12 000 cm^−1^, 17 500 cm^−1^, and 22 500 cm^−1^. The lack of an intense transition at *ca.* 18 000 cm^−1^ (*ε* = *ca.* 15 mM^−1^ cm^−1^), which is diagnostic for the formation of a metal-oxidized [Cu^III^Sal^Ph^]^+^ species,^21^ supports the formation of a ligand-oxidized species for [Cu(Sal)^Fc^]^3+^ instead. Further spectroscopic investigation of the electronic structure of [Cu(Sal)^Fc^]^3+^ is currently underway.

## Summary

In this paper, we report the synthesis, spectroscopic characterization and electrochemical studies of M(Sal)^Fc^, the Ni^II^ and Cu^II^ complexes of a novel Sal ligand bearing two ferrocene moieties in its secondary coordination sphere. The cyclic voltammograms of M(Sal)^Fc^ both exhibit an additional two-electron wave in comparison to its M(Sal)^Ph^ counterpart, which is assigned to the sequential oxidation of the two ferrocene moieties. The minimal separation between these two waves suggests the ferrocene moieties are incapable of electronic communication under these experimental conditions, consistent with their connection *via* a non-conjugated linker. This was further confirmed *via* the chemical oxidation of M(Sal)^Fc^ monitored by low temperatures UV-vis spectroscopy, where no near IR transitions were observed upon the addition of one equivalent of oxidant. This supports the formation of a mixed valent Fe^II^Fe^III^ intermediate species upon one-electron oxidation, while the addition of a second equivalent of oxidant yields a bis(ferrocenium) species. The subsequent anodic waves occur at similar potentials to the anodic waves of M(Sal)^Ph^, suggesting the ferrocene moieties are indeed in the secondary coordination sphere of M(Sal)^Fc^ and does not affect the electronic structure of the M(Sal) core. Accordingly, the addition of a third equivalence of oxidant to Ni(Sal)^Fc^ yielded intense near-IR transitions that are indicative of the formation of a fully delocalized Sal-ligand radical (Sal˙), while the same addition to Cu(Sal)^Fc^ yielded a species that is currently under further spectroscopic investigation.

## Author contributions

R. S., W. V., K. T.: investigation, formal analysis, methodology, visualization, writing – review & editing. L. C.: conceptualization, funding acquisition, project administration, resources, supervision, writing – original draft.

## Conflicts of interest

There are no conflicts to declare.

## Supplementary Material

RA-013-D2RA07671C-s001
