# Supplementary material for: NiII and CuII complexes of a salen ligand bearing ferrocenes in its secondary coordination sphere
Source: RSC Adv. 2023 Mar 6;13(11):7293–9. doi: 10.1039/d2ra07671c (PMC9986886; doi:10.1039/d2ra07671c)
Supplement: RA-013-D2RA07671C-s001 [file RA-013-D2RA07671C-s001.pdf]

Electronic Supplementary Material (ESI) for RSC Advances.
This journal is © The Royal Society of Chemistry 2023

Ni^{II} and Cu^{II} complexes of a salen ligand bearing ferrocenes in its secondary coordination sphere

sphere

Supporting Information

Rachel Sailer, Warren VandeVen, Kaeden Teindl, Linus Chiang

Mass spectrometry

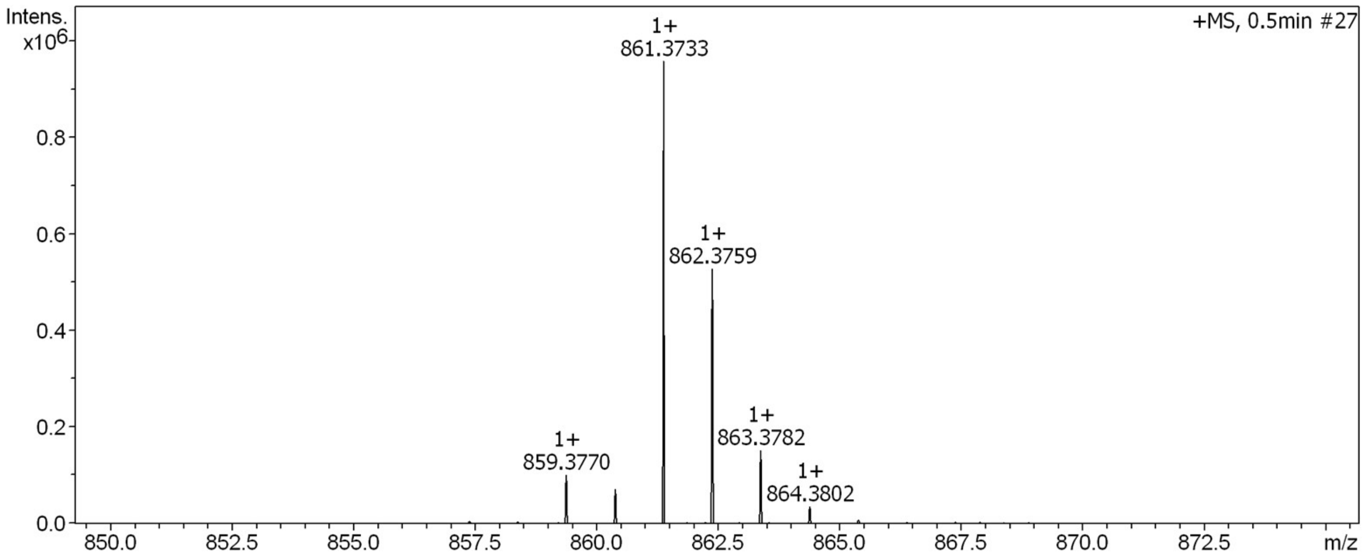

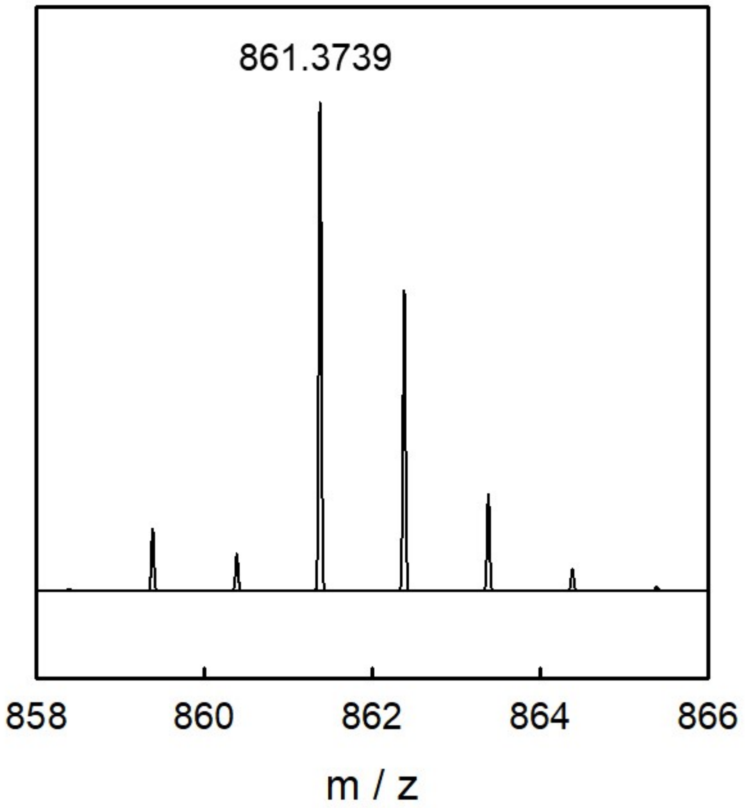

Figure S1. Experimental (top) and simulated (bottom) mass spectrum of $\mathbf{H_2(Sal)^{Fc}}$.

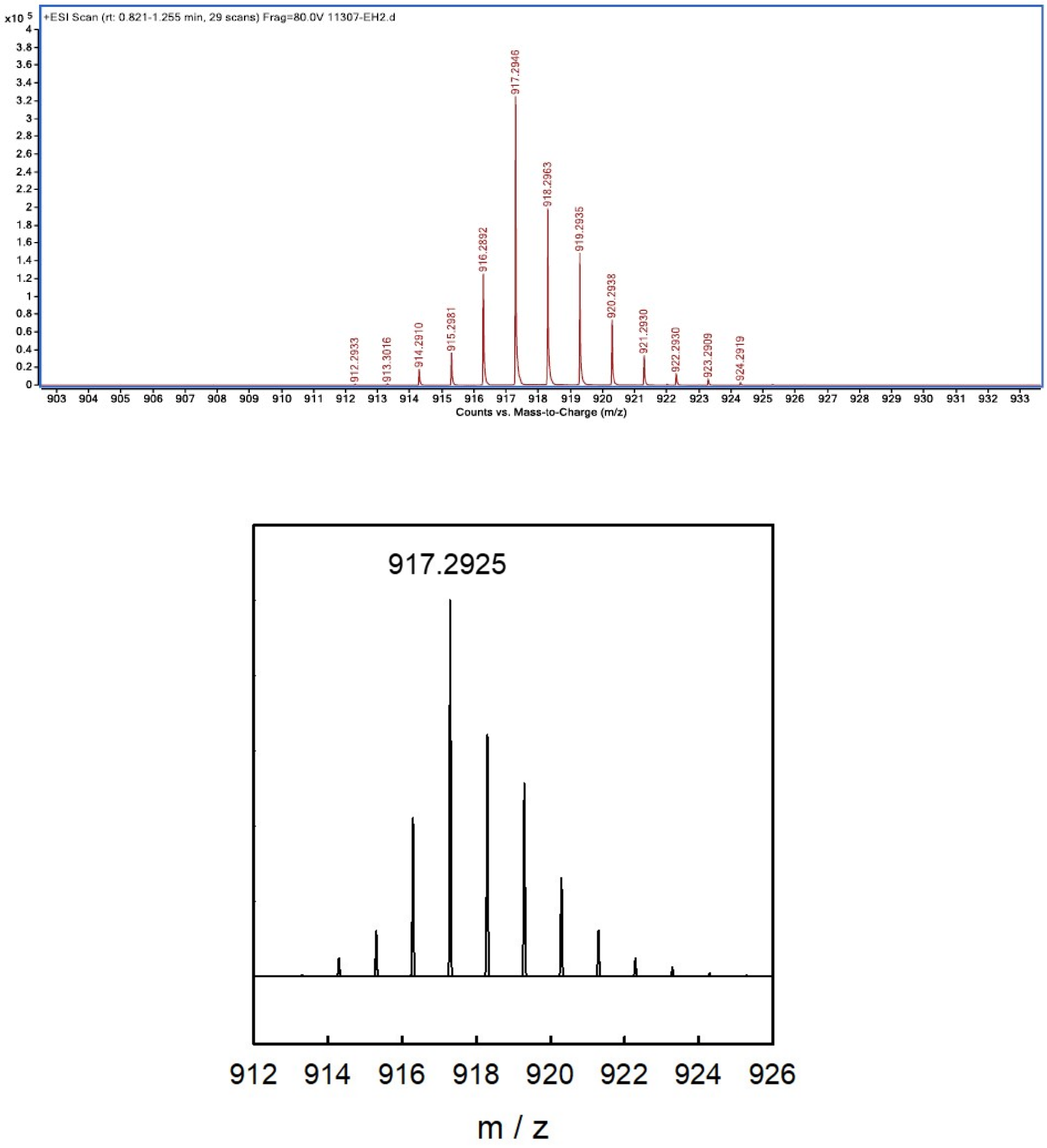

Figure S2. Experimental (top) and simulated (bottom) mass spectrum of **Ni(Sal)^{Fc}**.

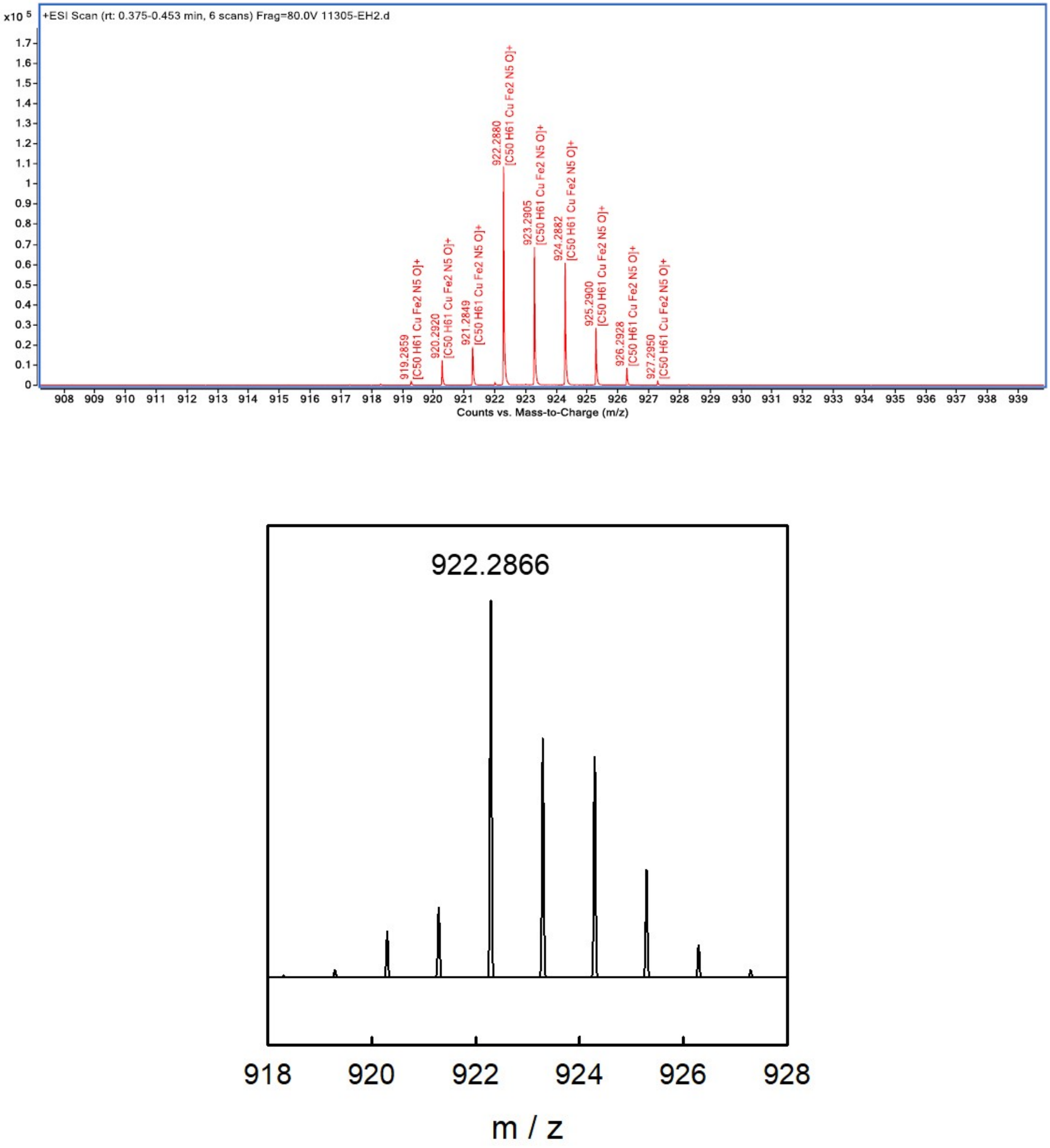

Figure S3. Experimental (top) and simulated (bottom) mass spectrum of **Cu(Sal)**$^{\mathbf{Fc}}$.

NMR spectroscopy:

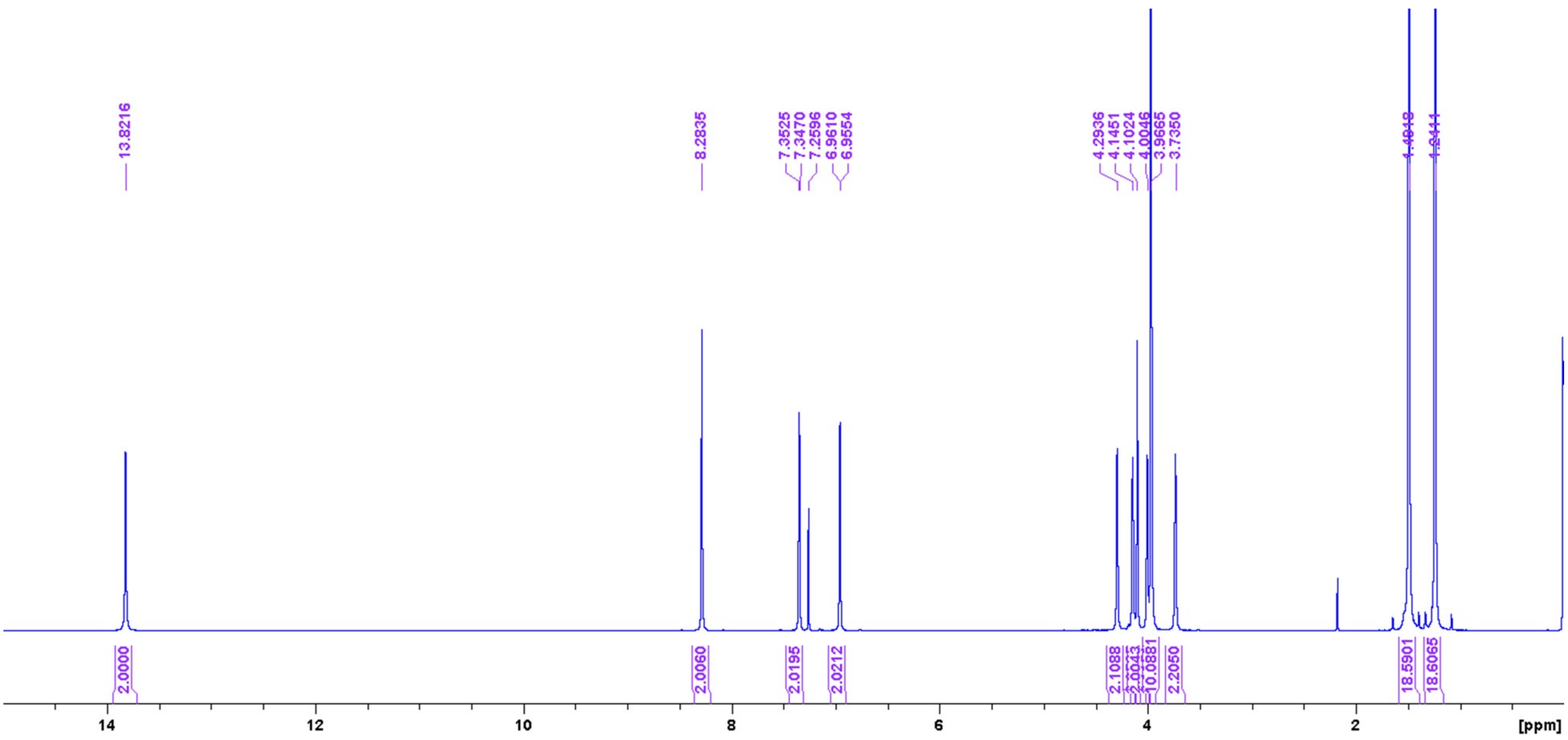

Figure S4. ^{1}H NMR spectrum of $\mathbf{H_2(Sal)^{Fc}}$ recorded in $CDCl_3$ at 298 K.

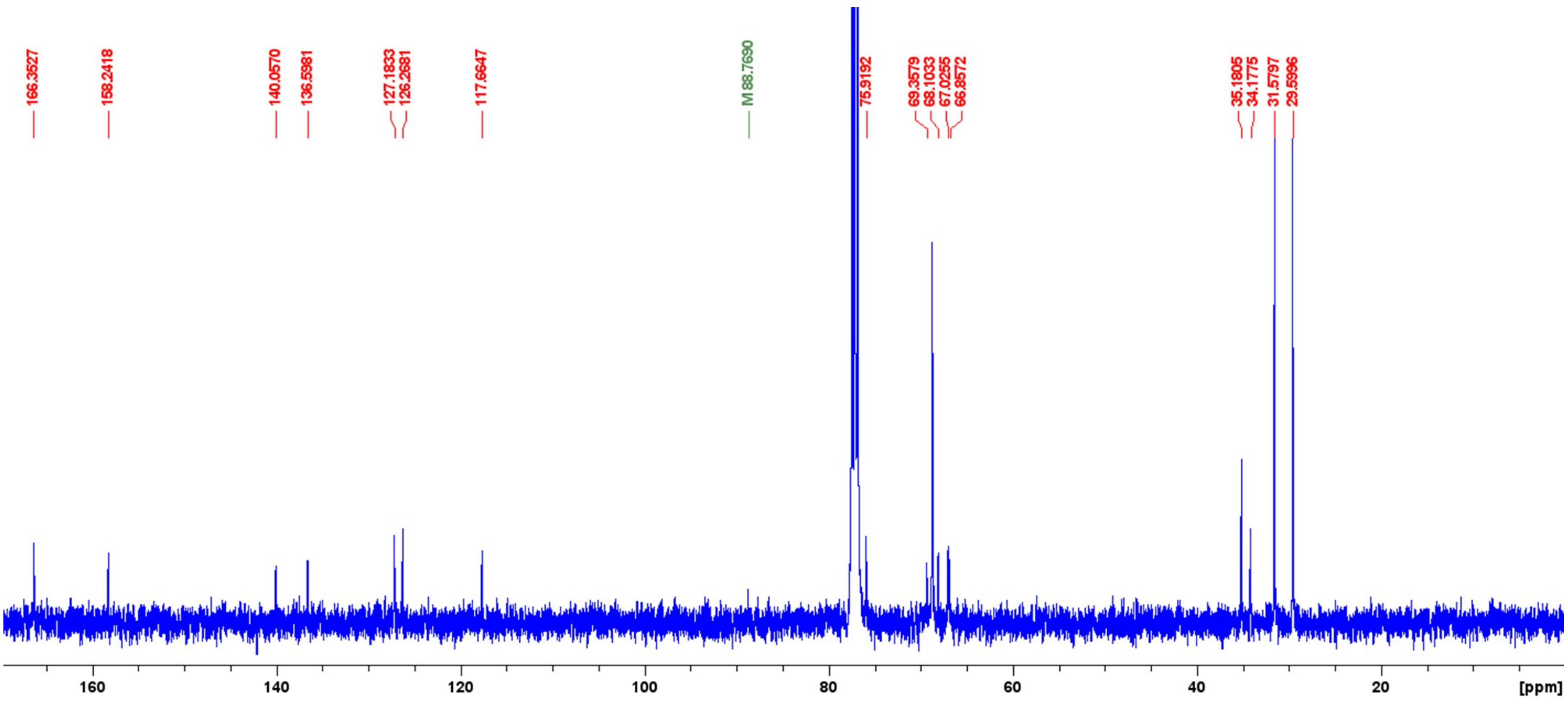

Figure S5. ^{13}C NMR spectrum of $\mathbf{H_2(Sal)^{Fc}}$ recorded in $CDCl_3$ at 298 K.

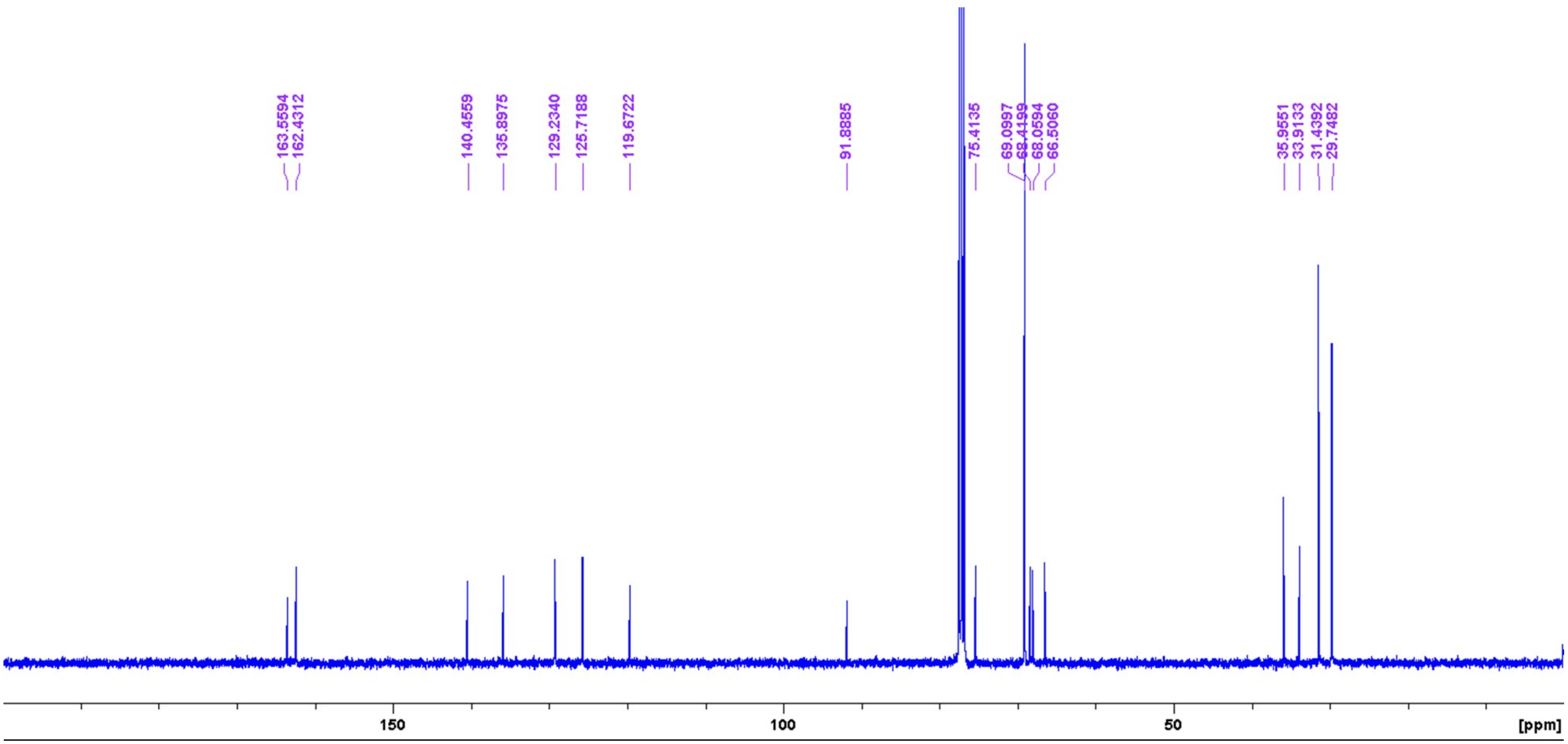

Figure S6. ^{13}C NMR spectrum of **Ni(Sal)^{Fc}** recorded in $CDCl_3$ at 298 K.

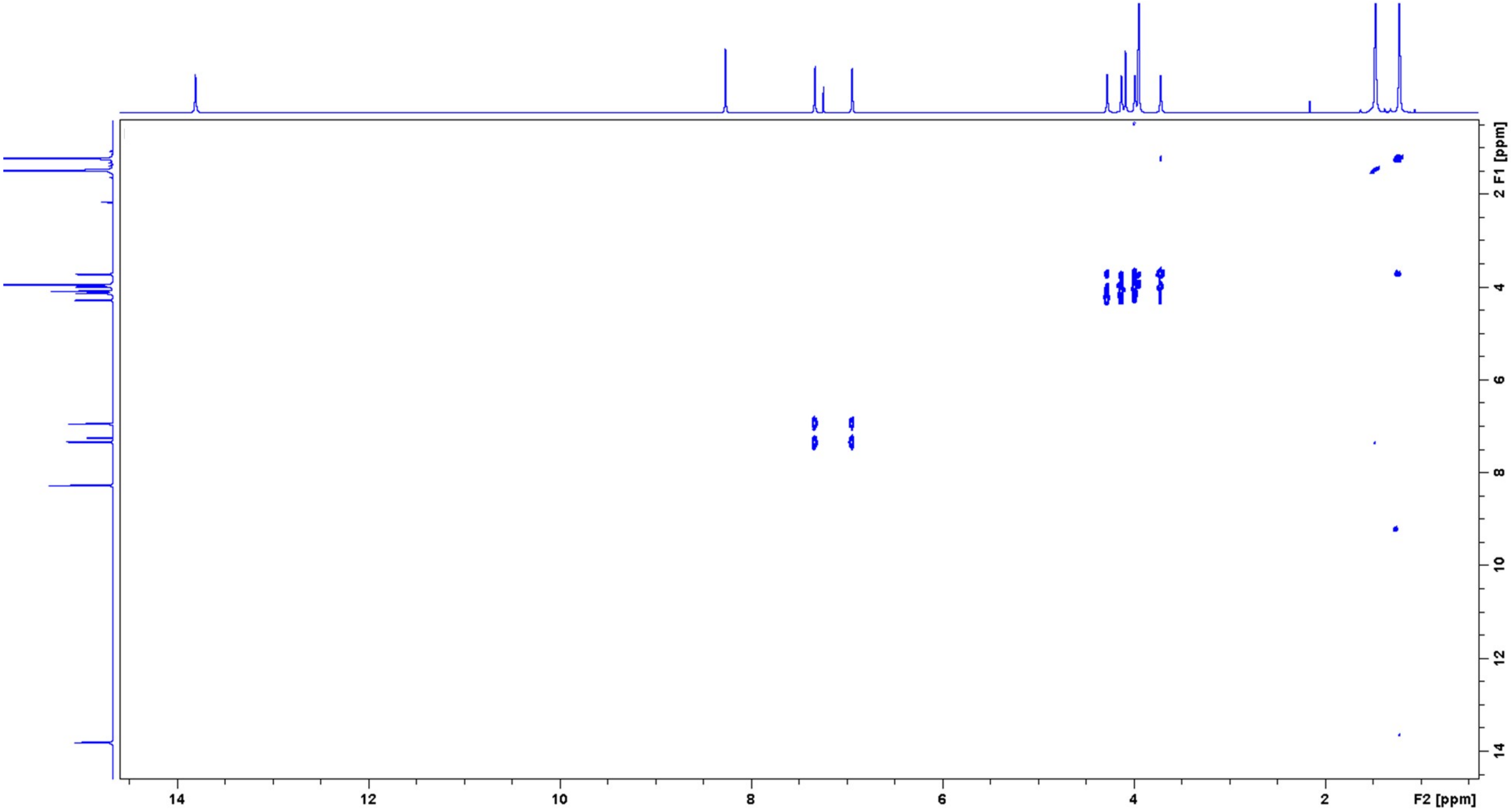

Figure S7. Full ^{1}H-^{1}H COSY spectrum of $\mathbf{H_2(Sal)^{Fc}}$ recorded in $CDCl_3$ at 298 K.

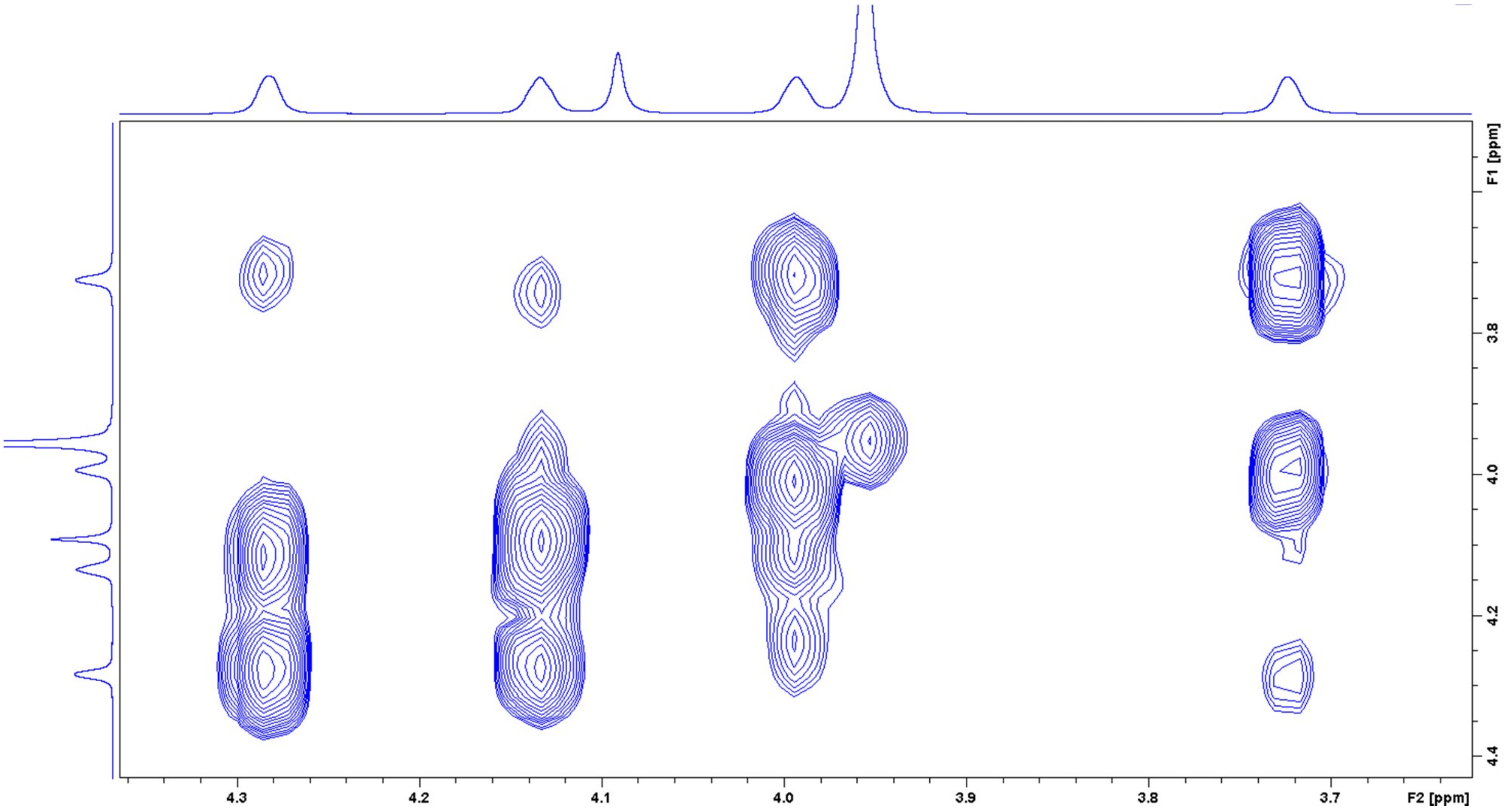

Figure S8. Magnified ^{1}H-^{1}H COSY spectrum of **$H_2(Sal)^{Fc}$** recorded in $CDCl_3$ at 298 K.

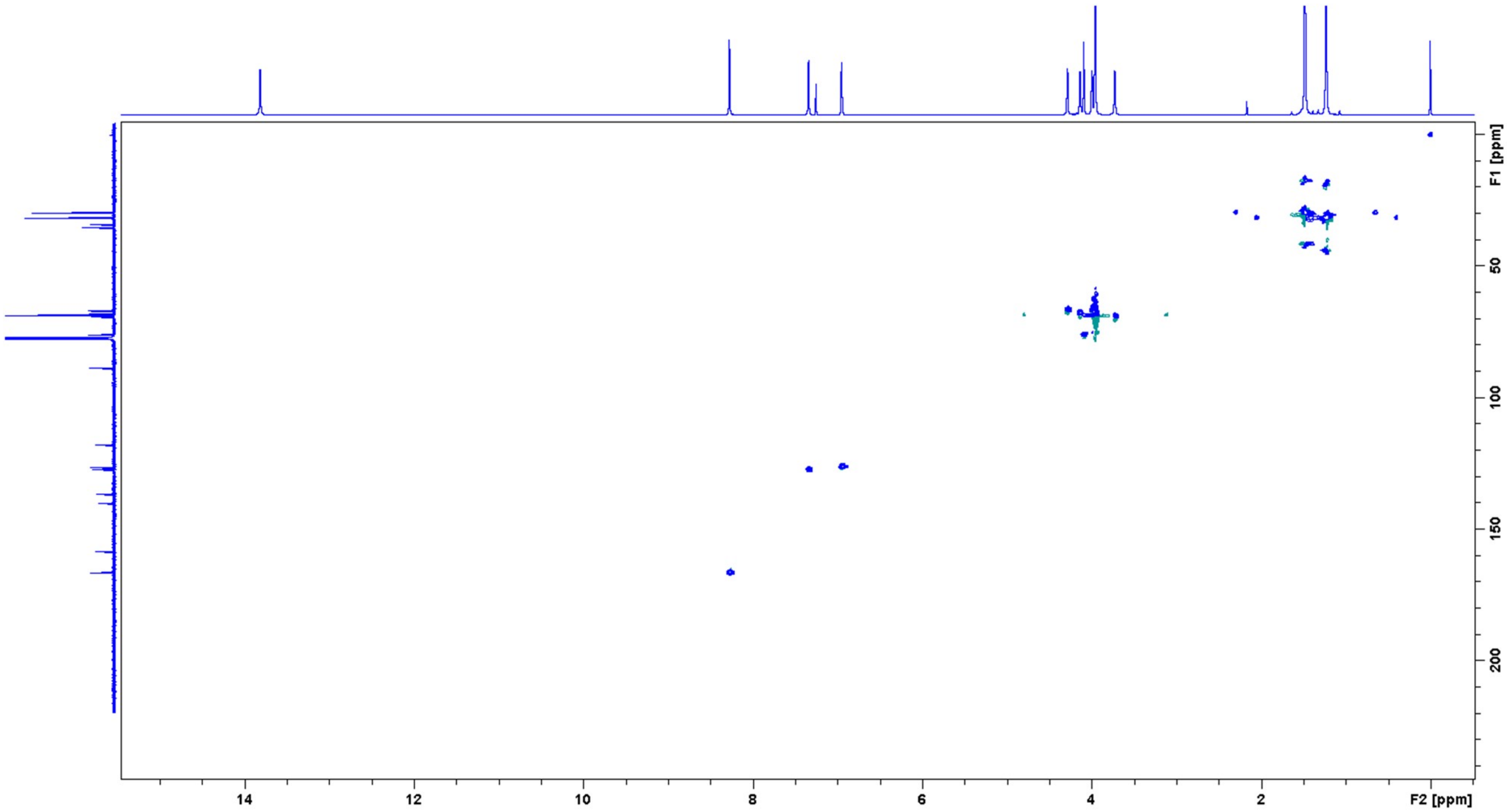

Figure S9. Full ^{1}H-^{13}C HSQC spectrum of $\mathbf{H_2(Sal)^{Fc}}$ recorded in $CDCl_3$ at 298 K.

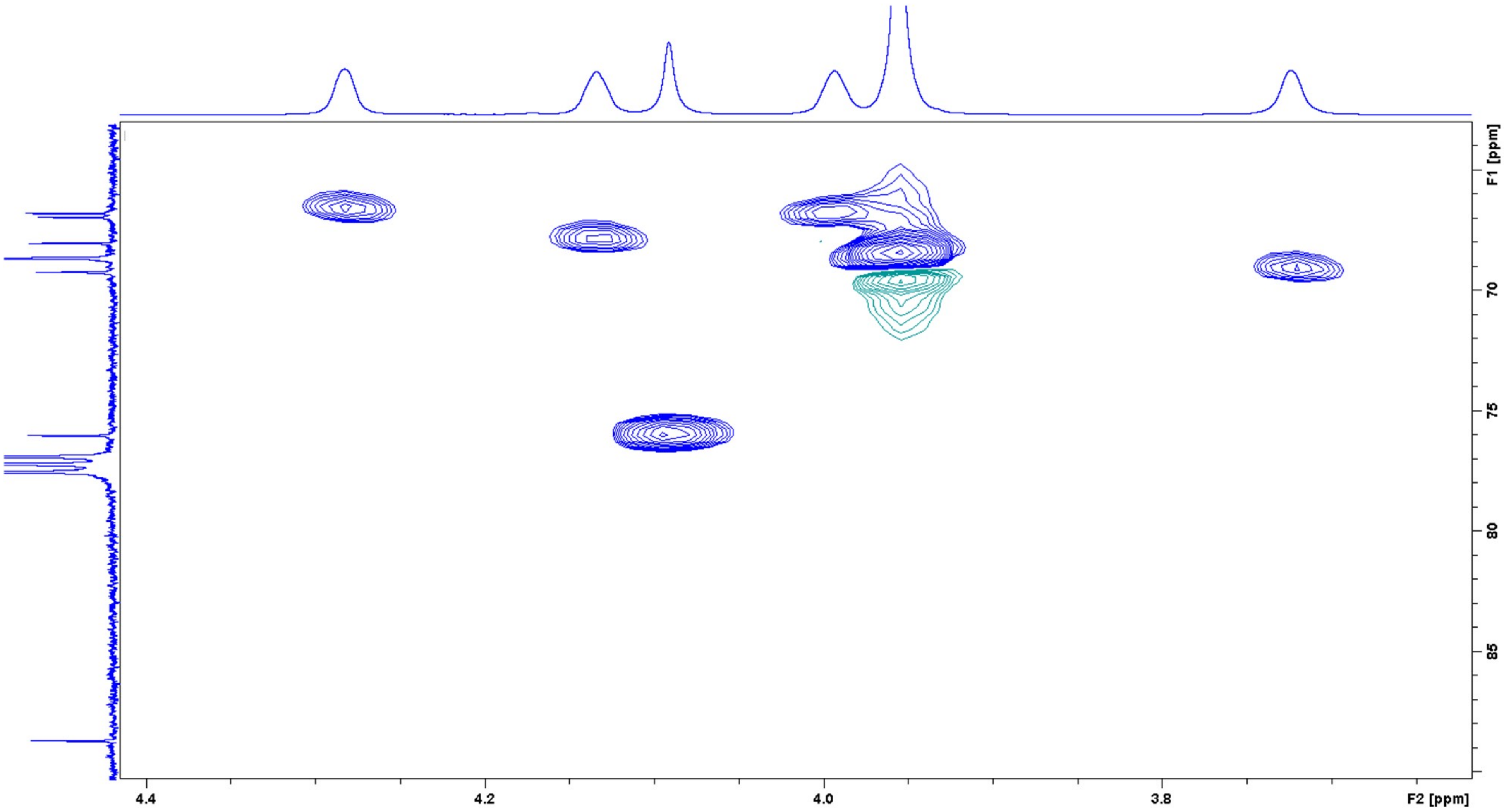

Figure S10. Magnified ^{1}H-^{13}C HSQC spectrum of **$H_2(Sal)^{Fc}$** recorded in $CDCl_3$ at 298 K.

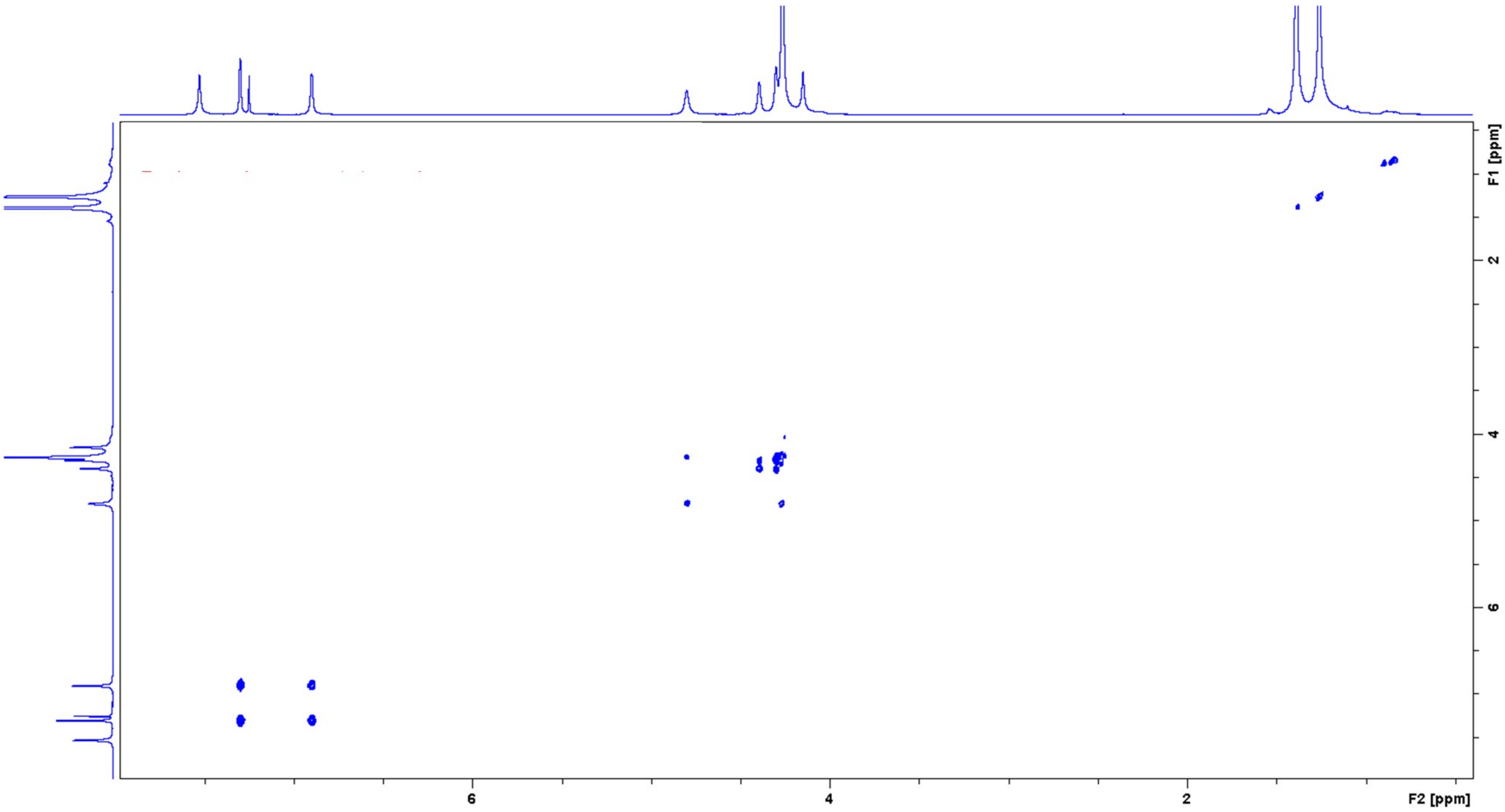

Figure S11. Full ^{1}H-^{1}H COSY spectrum of **Ni(Sal)^{Fc}** recorded in $CDCl_3$ at 298 K.

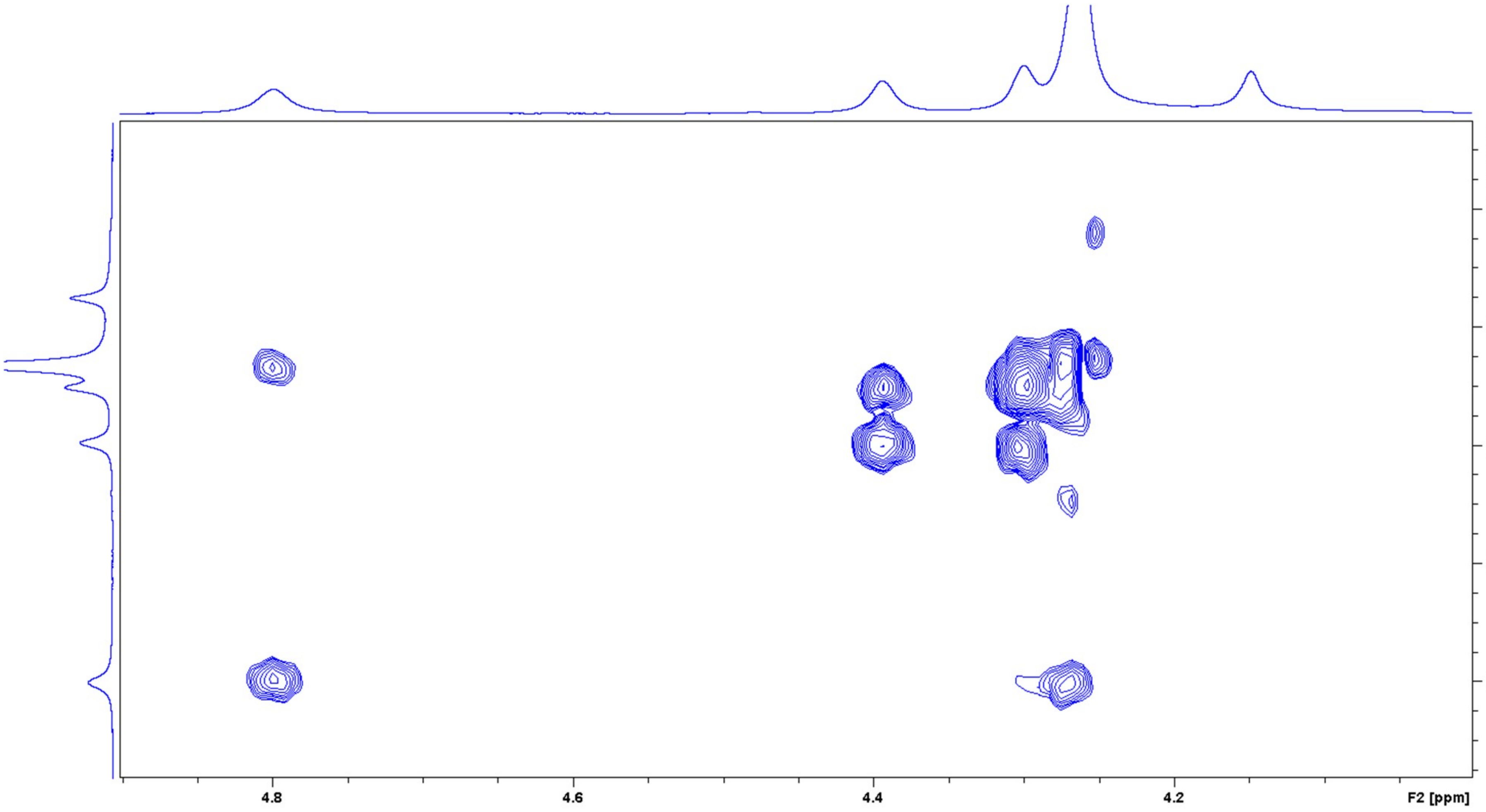

Figure S12. Magnified ^{1}H-^{1}H COSY spectrum of **Ni(Sal)^{Fc}** recorded in $CDCl_3$ at 298 K.

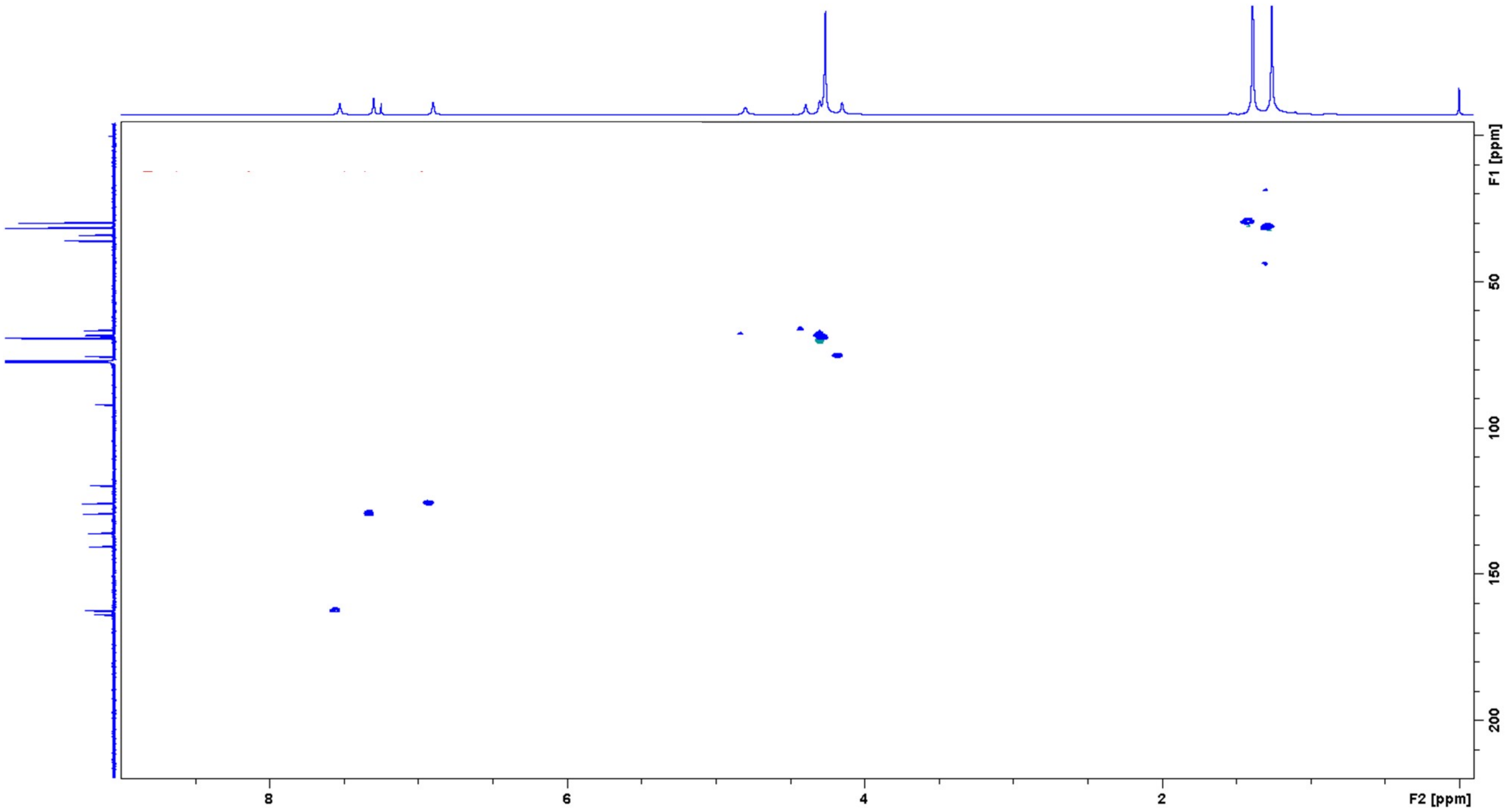

Figure S13. Full ^{1}H-^{13}C HSQC spectrum of **Ni(Sal)^{Fc}** recorded in $CDCl_3$ at 298 K.

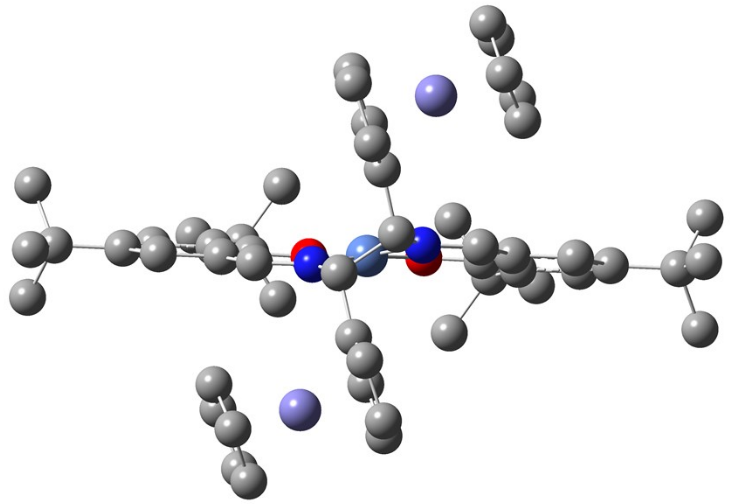

Figure S14. DFT optimized structure of **Ni(Sal)^{Fc}**. H-atoms have been omitted for clarity.

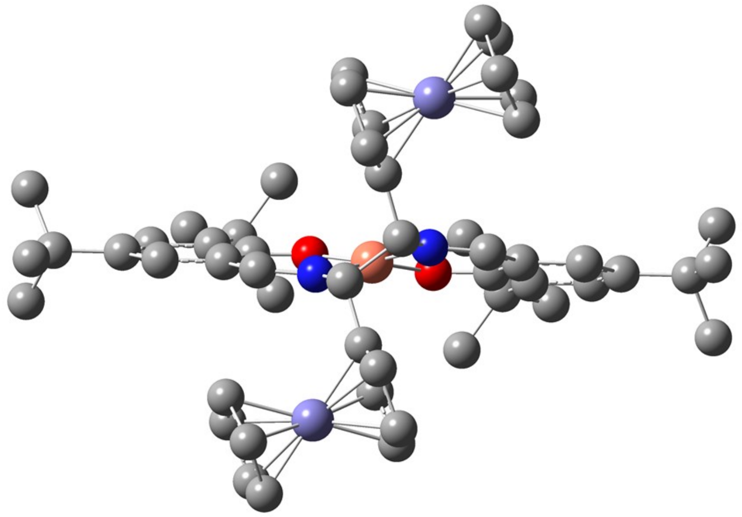

Figure S15. DFT optimized structure of **Cu(Sal)^{Fc}**. H-atoms have been omitted for clarity.

Table S1. Select DFT computed bond distances (Å). See experimental section for computational details.

Bond	**Ni(Sal)^{Fc}**	**Ni(Sal)^{Ph}**	**Cu(Sal)^{Fc}**	**Cu(Sal)^{Ph}**
M-N1	1.842	1.843	1.928	1.931
M -N2	1.842	1.843	1.928	1.930
M -O1	1.856	1.857	1.904	1.904
M -O2	1.856	1.857	1.904	1.905
C1-O1	1.309	1.309	1.306	1.305
C7-O2	1.308	1.309	1.306	1.305

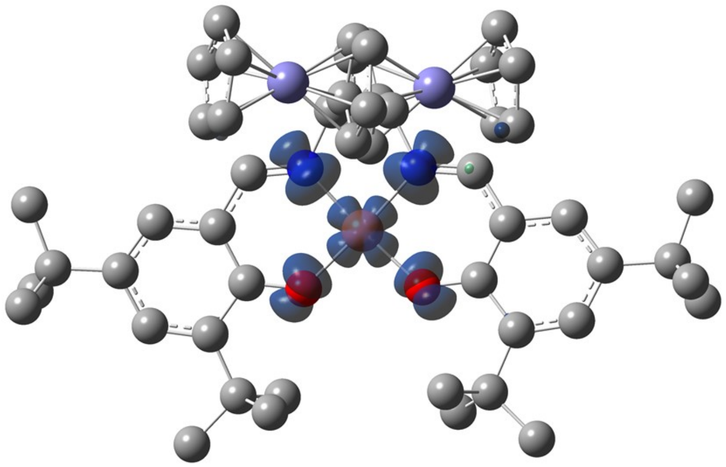

Figure S16. Spin density plot of **Cu(Sal)^{Fc}** (Isovalue: 0.0021).

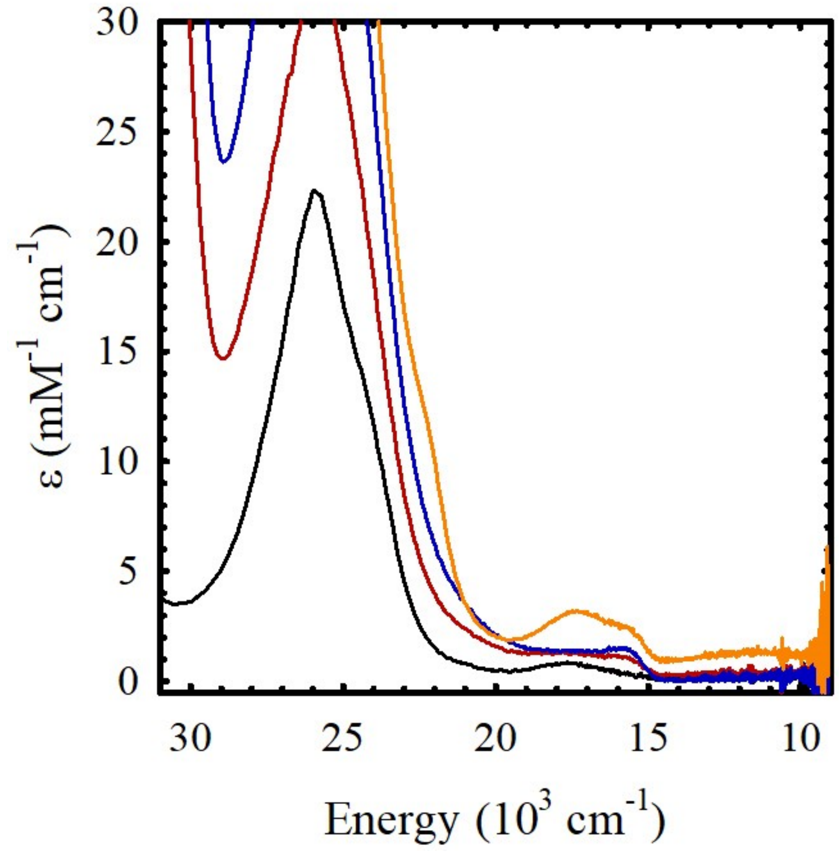

Figure S17. Sequential chemical oxidation of **Ni(Sal)^{Fc}** (black) to its one-electron oxidized (red), two-electron oxidized (blue), and three-electron oxidized (orange) species. Inset: Near-IR region. Conditions: 0.4 mM **Ni(Sal)^{Fc}** in CH_2Cl_2, 195 K.

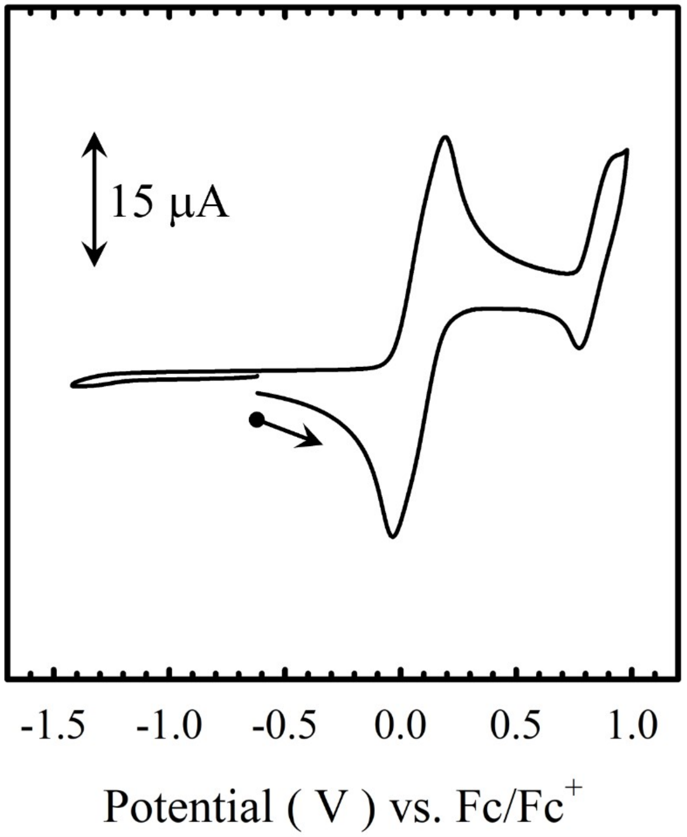

Figure S18. Cyclic voltammograms of *ca.* 1.5 mM solutions of $\mathbf{H_2(Sal)^{Fc}}$ in CH_2Cl_2.

Optimized XYZ Coordinates of neutral **M(Sal)^{Fc}** using the B3LYP functional with PCM = CH_2Cl_2.

**Ni(Sal)^{Fc}**				**Cu(Sal)^{Fc}**			
C(Fragment=2)	2.51651608	1.75600244	-0.62649469	C(Fragment=2)	2.59272581	1.77875997	-0.66002377
N(Fragment=2)	1.25656068	-0.82417873	-0.05347441	N(Fragment=2)	1.29832869	-0.79975320	0.06975184
O(Fragment=2)	1.25743653	1.85686547	-0.28486515	O(Fragment=2)	1.31621970	1.93058939	-0.43167836
C(Fragment=2)	4.62044815	2.76200595	-1.30086995	C(Fragment=2)	4.75350599	2.71272598	-1.26188692
H(Fragment=2)	5.19141297	3.64730587	-1.55306723	H(Fragment=2)	5.35611457	3.57291161	-1.52790857
C(Fragment=2)	5.30680033	1.52017716	-1.34744980	C(Fragment=2)	5.41570977	1.45885622	-1.19510566
O(Fragment=2)	-1.25716050	1.85704836	0.28434853	O(Fragment=2)	-1.31601488	1.93104640	0.42966699
C(Fragment=2)	4.56425506	0.40256431	-1.03412342	C(Fragment=2)	4.63563171	0.37457235	-0.85553947
H(Fragment=2)	5.00924951	-0.58769045	-1.05098986	H(Fragment=2)	5.06634021	-0.61978920	-0.78559332
C(Fragment=2)	2.51343359	-0.71698385	-0.36656403	C(Fragment=2)	2.55222367	-0.70370220	-0.24676439
H(Fragment=2)	3.10650266	-1.63350082	-0.39043547	H(Fragment=2)	3.14816799	-1.62227944	-0.26720929
C(Fragment=2)	3.19381208	0.49585480	-0.68004603	C(Fragment=2)	3.24850940	0.50226187	-0.58254865
C(Fragment=2)	7.35409588	0.04439562	-1.72756265	C(Fragment=2)	7.45207881	-0.07071400	-1.37078158
H(Fragment=2)	6.83125822	-0.60112993	-2.44277196	H(Fragment=2)	6.95657576	-0.74916230	-2.07489385
H(Fragment=2)	7.28050722	-0.41454649	-0.73484094	H(Fragment=2)	7.31563014	-0.46845454	-0.35848821
H(Fragment=2)	8.41341083	0.05890885	-2.00789930	H(Fragment=2)	8.52519931	-0.09008875	-1.59214421
C(Fragment=2)	6.79413808	1.47926225	-1.73690306	C(Fragment=2)	6.92164736	1.36999463	-1.49557538
C(Fragment=2)	6.97620913	2.05738315	-3.16082293	C(Fragment=2)	7.19490994	1.85936838	-2.93804836
H(Fragment=2)	6.41763582	1.46699569	-3.89649105	H(Fragment=2)	6.66677194	1.23573376	-3.66877993
H(Fragment=2)	8.03529786	2.04339854	-3.44658476	H(Fragment=2)	8.26786449	1.81078584	-3.16150291
H(Fragment=2)	6.62789805	3.09366800	-3.22853053	H(Fragment=2)	6.87177267	2.89546825	-3.08606149
C(Fragment=2)	7.61822311	2.32373440	-0.73567319	C(Fragment=2)	7.70501739	2.25867038	-0.49988117
H(Fragment=2)	8.68063158	2.31143934	-1.00889814	H(Fragment=2)	8.78060793	2.21291697	-0.71088576
H(Fragment=2)	7.52408904	1.92564160	0.28138768	H(Fragment=2)	7.54623003	1.92321149	0.53165132
H(Fragment=2)	7.29225538	3.36932038	-0.71789748	H(Fragment=2)	7.39915490	3.30865480	-0.56160994
C(Fragment=2)	2.63962432	4.33293924	-0.97095097	C(Fragment=2)	2.78120769	4.32923055	-1.11721423
C(Fragment=2)	2.10363706	4.67495568	0.43927159	C(Fragment=2)	2.18889524	4.74348220	0.25193491
H(Fragment=2)	1.62645069	5.66317819	0.43226234	H(Fragment=2)	1.73519106	5.74014623	0.17974765
H(Fragment=2)	2.92555483	4.70503608	1.16558972	H(Fragment=2)	2.97626350	4.78826502	1.01468522
H(Fragment=2)	1.37211756	3.93931029	0.77334796	H(Fragment=2)	1.42527989	4.03840995	0.58264041
C(Fragment=2)	3.63723231	5.44306987	-1.36108604	C(Fragment=2)	3.81747420	5.40248764	-1.50982032
H(Fragment=2)	4.04190590	5.30310641	-2.37030044	H(Fragment=2)	4.26582252	5.20721273	-2.49108759
H(Fragment=2)	4.47711335	5.51141825	-0.65969982	H(Fragment=2)	4.62526329	5.48822219	-0.77353801
H(Fragment=2)	3.11880412	6.40859864	-1.34706489	H(Fragment=2)	3.31929622	6.37712524	-1.56484581
C(Fragment=2)	1.49004179	4.36578477	-2.00684841	C(Fragment=2)	1.67440414	4.34438031	-2.19993410
H(Fragment=2)	1.00443700	5.34970359	-2.00299387	H(Fragment=2)	1.22709690	5.34427132	-2.26651759
H(Fragment=2)	0.73743077	3.60788935	-1.78629955	H(Fragment=2)	0.88614259	3.62611970	-1.97023247
H(Fragment=2)	1.87898084	4.18718278	-3.01706845	H(Fragment=2)	2.09384234	4.09881959	-3.18355963
C(Fragment=2)	0.72052238	-2.15255598	0.28942984	C(Fragment=2)	0.69922285	-2.10646496	0.35703421
C(Fragment=2)	-0.72074410	-2.15245376	-0.28952829	C(Fragment=2)	-0.69968225	-2.10644153	-0.35708701
N(Fragment=2)	-1.25658760	-0.82401038	0.05344796	N(Fragment=2)	-1.29857215	-0.79957159	-0.07014676
C(Fragment=2)	3.28471291	2.93209284	-0.96380213	C(Fragment=2)	3.40373084	2.91992875	-1.01603492
C(Fragment=2)	-2.51341727	-0.71664227	0.36659527	C(Fragment=2)	-2.55231680	-0.70325637	0.24687771
H(Fragment=2)	-3.10659103	-1.63308473	0.39055322	H(Fragment=2)	-3.14835402	-1.62176062	0.26795342
C(Fragment=2)	-3.19361209	0.49630771	0.68007653	C(Fragment=2)	-3.24831116	0.50292587	0.58249211
C(Fragment=2)	-4.56401360	0.40320396	1.03435345	C(Fragment=2)	-4.63528942	0.37548978	0.85632021
H(Fragment=2)	-5.00912670	-0.58699691	1.05129640	H(Fragment=2)	-5.06614113	-0.61885412	0.78700722
C(Fragment=2)	-5.30638302	1.52093141	1.34768134	C(Fragment=2)	-5.41506735	1.45998828	1.19589395
C(Fragment=2)	-4.61989945	2.76268225	1.30083086	C(Fragment=2)	-4.75268841	2.71380784	1.26183682
H(Fragment=2)	-5.19072780	3.64807091	1.55302291	H(Fragment=2)	-5.35504621	3.57416759	1.52786477
C(Fragment=2)	-3.28421115	2.93258832	0.96348669	C(Fragment=2)	-3.40303055	2.92077103	1.01514236
C(Fragment=2)	-2.51618859	1.75636346	0.62625125	C(Fragment=2)	-2.59236058	1.77938871	0.65904907
C(Fragment=2)	-2.63897608	4.33337190	0.97030330	C(Fragment=2)	-2.78029104	4.33004147	1.11553496
C(Fragment=2)	-1.48927572	4.36626037	2.00606307	C(Fragment=2)	-1.67303327	4.34535234	2.19779848
H(Fragment=2)	-1.00348467	5.35008613	2.00192272	H(Fragment=2)	-1.22546632	5.34516531	2.26383934
H(Fragment=2)	-0.73682672	3.60817188	1.78561892	H(Fragment=2)	-0.88501993	3.62683948	1.96802347

H(Fragment=2)	-1.87813803	4.18796919	3.01636740	H(Fragment=2)	-2.09211225	4.10023372	3.18168491
C(Fragment=2)	-2.10311996	4.67508286	-0.44005056	C(Fragment=2)	-2.18846774	4.74378230	-0.25398728
H(Fragment=2)	-2.92512857	4.70512663	-1.16626665	H(Fragment=2)	-2.97613126	4.78841079	-1.01644235
H(Fragment=2)	-1.37171893	3.93930655	-0.77409694	H(Fragment=2)	-1.42508169	4.03847707	-0.58471206
H(Fragment=2)	-1.62583636	5.66325984	-0.43326622	H(Fragment=2)	-1.73463863	5.74042119	-0.18229737
C(Fragment=2)	-3.63641176	5.44368541	1.36034929	C(Fragment=2)	-3.81624765	5.40355034	1.50823324
H(Fragment=2)	-3.11788706	6.40915883	1.34604168	H(Fragment=2)	-3.31793302	6.37815259	1.56266362
H(Fragment=2)	-4.04094656	5.30398923	2.36965627	H(Fragment=2)	-4.26416744	5.20870386	2.48978152
H(Fragment=2)	-4.47639202	5.51196351	0.65907553	H(Fragment=2)	-4.62436522	5.48909141	0.77228819
C(Fragment=2)	-6.79365764	1.48022890	1.73738721	C(Fragment=2)	-6.92084825	1.37140005	1.49722858
C(Fragment=2)	-7.35376546	0.04541892	1.72831730	C(Fragment=2)	-7.45156800	-0.06924462	1.37292350
H(Fragment=2)	-8.41302506	0.06007007	2.00885595	H(Fragment=2)	-8.52456557	-0.08843053	1.59489760
H(Fragment=2)	-6.83085346	-0.60007892	2.44349721	H(Fragment=2)	-6.95576961	-0.74767887	2.07684142
H(Fragment=2)	-7.28041472	-0.41364277	0.73563308	H(Fragment=2)	-7.31575270	-0.46713484	0.36060382
C(Fragment=2)	-6.97542505	2.05854469	3.16126682	C(Fragment=2)	-7.19321287	1.86100686	2.93979484
H(Fragment=2)	-6.62698067	3.09479663	3.22878912	H(Fragment=2)	-6.86986906	2.89709043	3.08746787
H(Fragment=2)	-6.41680089	1.46818154	3.89691593	H(Fragment=2)	-6.66471805	1.23741289	3.67030226
H(Fragment=2)	-8.03446827	2.04471984	3.44720471	H(Fragment=2)	-8.26604253	1.81257778	3.16388304
C(Fragment=2)	-7.61782718	2.32466521	0.73619496	C(Fragment=2)	-7.70465522	2.26005967	0.50186831
H(Fragment=2)	-7.29176044	3.37021717	0.71824345	H(Fragment=2)	-7.39859889	3.31000716	0.56327244
H(Fragment=2)	-8.68019078	2.31250417	1.00959933	H(Fragment=2)	-8.78012945	2.21450195	0.71350744
H(Fragment=2)	-7.52390145	1.92644408	-0.28083491	H(Fragment=2)	-7.54652436	1.92443553	-0.52971138
Ni(Fragment=1)	0.00007917	0.52105666	-0.00008736	Cu(Fragment=1)	0.00001179	0.62427509	-0.00054356
C(Fragment=2)	-0.71488746	-2.41888758	-1.77785871	C(Fragment=2)	-0.54908904	-2.35382644	-1.84081989
C(Fragment=2)	-0.59807691	-3.72435041	-2.35968757	C(Fragment=2)	-0.32915761	-3.64258096	-2.42969619
C(Fragment=2)	-0.65874611	-1.46899022	-2.84710367	C(Fragment=2)	-0.45913644	-1.38491857	-2.89150953
C(Fragment=2)	-0.47112681	-3.57710605	-3.77340087	C(Fragment=2)	-0.10759811	-3.46640802	-3.82862761
H(Fragment=2)	-0.61576618	-4.66090650	-1.81576032	H(Fragment=2)	-0.34357869	-4.58820779	-1.90160700
C(Fragment=2)	-0.51278690	-2.18246977	-4.07407790	C(Fragment=2)	-0.19132207	-2.07079482	-4.11365188
H(Fragment=2)	-0.74427270	-0.39599231	-2.74288878	H(Fragment=2)	-0.60468006	-0.31850677	-2.78377348
H(Fragment=2)	-0.39102717	-4.38288896	-4.49181736	H(Fragment=2)	0.06158384	-4.25585563	-4.54970034
H(Fragment=2)	-0.47007044	-1.74108921	-5.06149780	H(Fragment=2)	-0.09745875	-1.61188680	-5.08944637
C(Fragment=2)	0.71452499	-2.41905459	1.77775598	C(Fragment=2)	0.54855738	-2.35350161	1.84081345
C(Fragment=2)	0.59785919	-3.72456324	2.35951744	C(Fragment=2)	0.32851080	-3.64211675	2.42994775
C(Fragment=2)	0.65795023	-1.46920891	2.84702853	C(Fragment=2)	0.45840402	-1.38435223	2.89125787
C(Fragment=2)	0.47060283	-3.57739910	3.77321419	C(Fragment=2)	0.10668711	-3.46562475	3.82879364
H(Fragment=2)	0.61588357	-4.66109654	1.81556127	H(Fragment=2)	0.34303661	-4.58786121	1.90207030
C(Fragment=2)	0.51192135	-2.18276243	4.07395222	C(Fragment=2)	0.19036305	-2.06994114	4.11351313
H(Fragment=2)	0.74329524	-0.39619043	2.74287014	H(Fragment=2)	0.60394564	-0.31796198	2.78329486
H(Fragment=2)	0.39048321	-4.38322188	4.49158390	H(Fragment=2)	-0.06262391	-4.25490555	4.55001890
H(Fragment=2)	0.46887720	-1.74142317	5.06137676	H(Fragment=2)	0.09632265	-1.61080613	5.08918393
Fe(Fragment=2)	-2.23548485	-2.75972247	-3.12332667	Fe(Fragment=2)	-1.94994651	-2.73954191	-3.29611529
Fe(Fragment=2)	2.23488813	-2.75960362	3.12349250	Fe(Fragment=2)	1.94914382	-2.73888807	3.29648595
C(Fragment=2)	3.70622334	-3.30568992	4.44925674	C(Fragment=2)	3.29676298	-3.32859185	4.73113774
C(Fragment=2)	3.81618145	-4.06711591	3.24665543	C(Fragment=2)	3.45637387	-4.11334576	3.54964779
C(Fragment=2)	3.83397235	-1.92455301	4.11096041	C(Fragment=2)	3.51030930	-1.96008721	4.38489277
H(Fragment=2)	3.53253469	-3.70464797	5.44047040	H(Fragment=2)	3.03580680	-3.70330168	5.71263441
C(Fragment=2)	4.01151376	-3.15619333	2.16463990	C(Fragment=2)	3.76811737	-3.22967970	2.47186594
H(Fragment=2)	3.74470970	-5.14425264	3.16618566	H(Fragment=2)	3.34179783	-5.18741918	3.47851289
C(Fragment=2)	4.02284281	-1.83093952	2.69935405	C(Fragment=2)	3.80196132	-1.89775380	2.98892004
H(Fragment=2)	3.77536409	-1.09229302	4.80054204	H(Fragment=2)	3.44107901	-1.11511120	5.05784487
H(Fragment=2)	4.12514883	-3.42738284	1.12281373	H(Fragment=2)	3.94014046	-3.52155112	1.44368012
H(Fragment=2)	4.14032412	-0.91535776	2.13466473	H(Fragment=2)	4.00007999	-0.99762459	2.42182434
C(Fragment=2)	-3.70688767	-3.30680016	-4.44860770	C(Fragment=2)	-3.29779510	-3.33030260	-4.73012615
C(Fragment=2)	-3.81664067	-4.06753397	-3.24554273	C(Fragment=2)	-3.45738624	-4.11402486	-3.54795550
C(Fragment=2)	-3.83475749	-1.92547201	-4.11111725	C(Fragment=2)	-3.51111211	-1.96147197	-4.38502790
H(Fragment=2)	-3.53327089	-3.70633374	-5.43960144	H(Fragment=2)	-3.03699788	-3.70588257	-5.71133231
C(Fragment=2)	-4.01199802	-3.15599802	-2.16405630	C(Fragment=2)	-3.76889051	-3.22939728	-2.47089052
H(Fragment=2)	-3.74504679	-5.14461476	-3.16445232	H(Fragment=2)	-3.34295864	-5.18805418	-3.47592065
C(Fragment=2)	-4.02352008	-1.83105497	-2.69954773	C(Fragment=2)	-3.80260065	-1.89790193	-2.98907422

H(Fragment=2)	-3.77633291	-1.09362184	-4.80120760	H(Fragment=2)	-3.44183603	-1.11708417	-5.05871311
H(Fragment=2)	-4.12554313	-3.42646007	-1.12203084	H(Fragment=2)	-3.94086743	-3.52037033	-1.44244426
H(Fragment=2)	-4.14108075	-0.91517531	-2.13535413	H(Fragment=2)	-4.00051506	-0.99726481	-2.42271155
H(Fragment=2)	-1.30613812	-2.93105754	0.21185319	H(Fragment=2)	-1.30496313	-2.90904570	0.08146075
H(Fragment=2)	1.30583254	-2.93122883	-0.21194428	H(Fragment=2)	1.30437743	-2.90926674	-0.08132443
